# Retinol and retinol binding protein 4 levels and COVID-19: a Mendelian randomization study

**DOI:** 10.1186/s12890-024-03013-w

**Published:** 2024-04-26

**Authors:** Haixia Wang, Zhiyun Zhang, Li Xie, Kongli Lu, Shuyi Zhang, Shunpeng Xing

**Affiliations:** 1 Department of Critical Care Medicine, Renji Hospital, School of Medicine, Shanghai Jiao Tong University, 200127 Shanghai, China; 2 Clinical Research Institute, School of Medicine, Shanghai Jiao Tong University, 200025 Shanghai, China; 3 Department of Cardiovascular Surgery, Renji Hospital, School of Medicine, Shanghai Jiao Tong University, 200127 Shanghai, China

**Keywords:** COVID-19, Retinol, Vitamin A, Mendelian randomization

## Abstract

**Background:**

The Corona Virus Disease 2019 (COVID-19) pandemic has struck globally. Whether the related proteins of retinoic acid (RA) signaling pathway are causally associated with the risk of COVID-19 remains unestablished. We conducted a two-sample Mendelian randomization (MR) study to assess the associations of retinol, retinol binding protein 4 (RBP4), retinol dehydrogenase 16 (RDH16) and cellular retinoic acid binding protein 1 (CRABP1) with COVID-19 in European population.

**Methods:**

The outcome utilized the summary statistics of COVID-19 from the COVID-19 Host Genetics Initiative. The exposure data were obtained from public genome wide association study (GWAS) database. We extracted SNPs from exposure data and outcome data. The inverse variance weighted (IVW), MR-Egger and Wald ratio methods were employed to assess the causal relationship between exposure and outcome. Sensitivity analyses were performed to ensure the validity of the results.

**Results:**

The MR estimates showed that retinol was associated with lower COVID-19 susceptibility using IVW (OR: 0.69, 95% CI: 0.53–0.90, P: 0.0065), whereas the associations between retinol and COVID-19 hospitalization or severity were not significant. RBP4 was associated with lower COVID-19 susceptibility using the Wald ratio (OR: 0.83, 95% CI: 0.72–0.95, P: 0.0072). IVW analysis showed RDH16 was associated with increased COVID-19 hospitalization (OR: 1.10, 95% CI: 1.01–1.18, P: 0.0199). CRABP1 was association with lower COVID-19 susceptibility (OR: 0.95, 95% CI: 0.91–0.99, P: 0.0290) using the IVW.

**Conclusions:**

We found evidence of possible causal association of retinol, RBP4, RDH16 and CRABP1 with the susceptibility, hospitalization and severity of COVID-19. Our study defines that retinol is significantly associated with lower COVID-19 susceptibility, which provides a reference for the prevention of COVID-19 with vitamin A supplementation.

**Supplementary Information:**

The online version contains supplementary material available at 10.1186/s12890-024-03013-w.

## Background

The coronavirus disease 2019 (COVID-19) pandemic caused by severe acute respiratory syndrome coronavirus 2 (SARS-CoV2) has struck globally and led to substantial morbidity and mortality [1]. The most common symptom of COVID-19 is pneumonia. Respiratory droplet is considered the primary way of transmission [2]. The pathogenesis of the disease is currently being extensively investigated, along with potential treatments. The main treatments are antiviral agents, anticoagulant treatments, steroids and immunomodulatory agents. However, the efficacy of many treatments is limited and controversial [1].

Approximately 90 years ago, vitamin A (VA) was known as “the anti-infective” vitamin [3]. VA deficiency reduces the host’s ability to fight infections, especially pneumonia [4]. In vivo, VA is converted to retinol and stored in the hepatic stellate cells [5]. Retinol is not biologically active. In the bloodstream, retinol is released and binds to retinol-binding protein 4 (RBP4). After entering the target cell, free retinol undergoes oxidation to give retinal, in the presence of retinol dehydrogenase (RDH) and subsequent oxidation by retinaldehyde dehydrogenases to retinoic acid (RA) [6]. The RA binding with cellular RA binding protein (CRABP) can not only allow it to enter the nucleus and induce genomic and non-genomic effects [7], but also inhibit or activate cytosolic kinase signaling [8]. However, RA will be rapidly metabolized by the cytochrome P450 enzymes, and its half-life is around one hour [9]. RA signaling pathway controls a wide range of physiological processes in numerous organs and is crucial for intact immune function. Disregulated retinoid signaling can cause serious illness, including embryonic developmental defects, diabetes, metabolic syndrome and acute promyelocytic leukemia [7].

An observational study examined that, compared to nonpatients, hospitalized COVID-19 patients had reduced VA plasma levels regardless of disease severity, and critically ill COVID-19 patients had reduced RBP4 plasma levels during their acute phase of illness [10]. Depletion of retinol due to the large amount of viral RNA and consequent overwhelming immune stimulation occurred during COVID-19 infection. Many researches proposed that retinol depletion and subsequent retinol signaling impairment played a crucial role in the pathogenesis of COVID-19 and its associated broad systemic effects [11–13].

However, these observational studies are prone to confounders. The impacts of retinol and RA signaling pathway on host susceptibility to COVID-19 and disease severity remains uncertain. We conducted a Mendelian Randomization (MR) to assess their potential impact on COVID-19. MR is a method that uses genetic variation to strengthen causal inference regarding modifiable exposures influencing risk of outcomes [14]. Here, we assessed the association between the development and severity of COVID-19, and retinol and RA signaling pathway, by MR, utilizing alleles as proxies for the genetically predicted circulating status of retinol, RBP4, RDH16 and CRABP1.

## Methods

### Study design and data sources

**Fig. 1:**
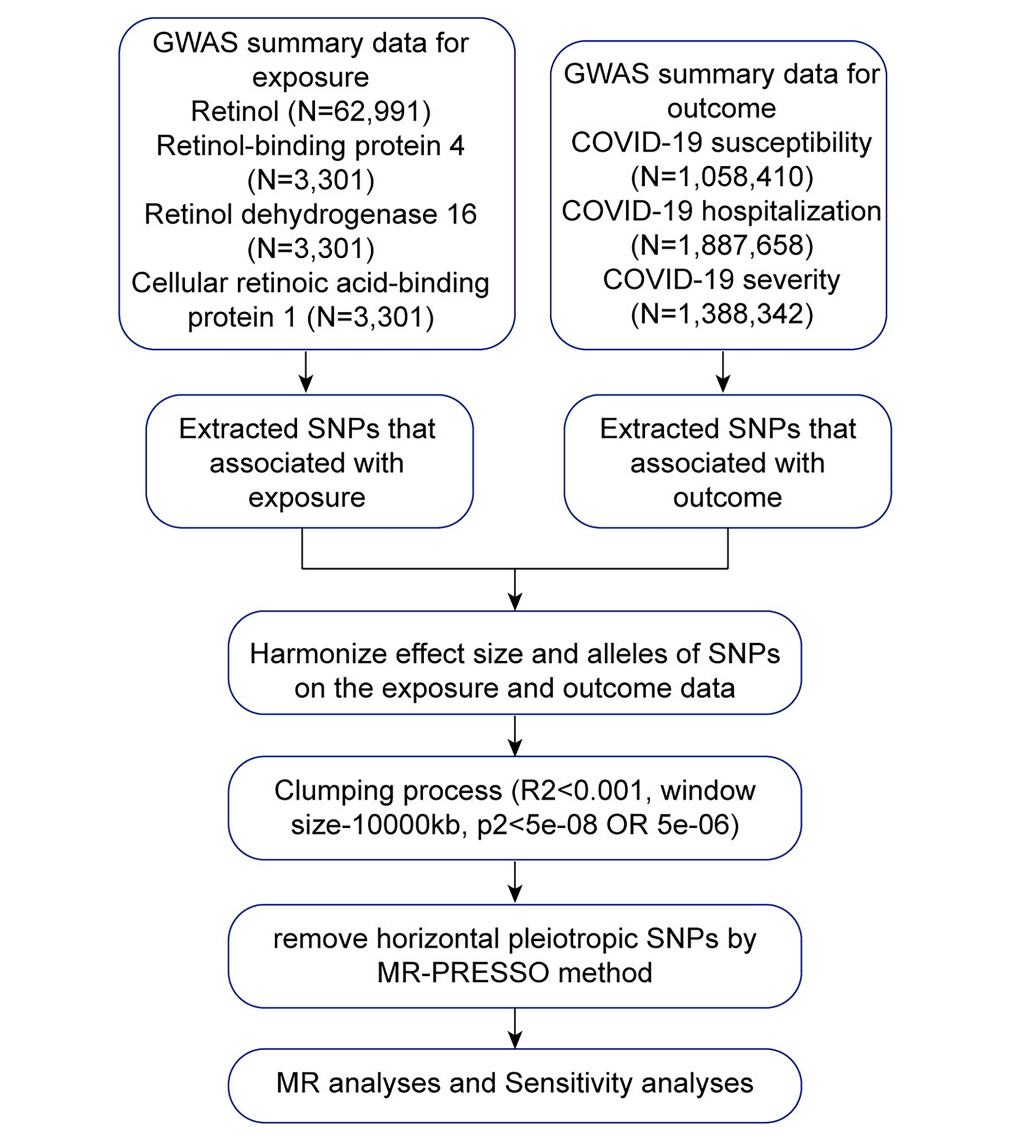
The diagram of the study design. GWAS, genome-wide association studies; MR-PRESSO, Mendelian Randomization Pleiotropy Residual Sum and Outlier; MR, mendelian randomization. R2, A measure of the linkage disequilibrium between two genetic loci to quantify their correlation (value of 1 denotes perfect correlation). SNP, single nucleotide polymorphism; Window size, the length of the region of linkage disequilibrium

The overall design of this study is shown in Fig. 1. We adopted the two-sample MR method to evaluate possible causal relationships between COVID-19 (outcome) and retinol, RBP4, RDH16 and CRABP1 (exposure). MR rests on three main assumptions: (1) the genetic variants selected as the instrumental variables (IVs) are robustly associated with the exposure; (2) the genetic variants are not associated with confounders that may affect the relationship between exposure and outcome; (3) genetic variants affect the outcome only through the exposure, not other pathways.

Single nucleotide polymorphisms (SNPs) for retinol in the European population were selected as IVs from IEU analysis of UK Biobank phenotypes. The summary-level data of RBP4, RDH16 and CRABP1 in the European population were extracted from the complete GWAS summary data on protein levels as described by Sun et al. 2018 [15]. Three outcomes related to COVID-19 were assessed in the study, including COVID-19 susceptibility, hospitalization and severity. The European-specific summary statistics were obtained from the COVID-19 host genetics initiative (https://www.covid19hg.org/results/) GWAS (Release 5) [16, 17], which provided genetic connections with COVID-19 phenotypes. These GWAS summary statistics are available at https://gwas.mrcieu.ac.uk/. The study design of the sample collection, quality control procedures, the phenotypes of GWAS and imputation methods have been described in the original publications. Further details regarding these summary statistics are provided in supplementary documents 1. The original GWAS had been approved by corresponding ethics committee.

### Selection of instrumental variables

SNPs associated with retinol and other associated proteins were identified at the genome-wide significance threshold (*P* < 5 × 10^− 8^), and independent SNPs without linkage disequilibrium (r^2^ < 0.001 and clump window > 10,000 kb) were used as IVs (supplementary documents 1). Due to the limited number of SNPs extracted for genome-wide significant variants, we also performed MR analysis with a more liberal cut-off of genetic predictors (*P* < 5 × 10^− 6^). Phenoscanner website [18] was used to examine the pleiotropic effects of selected IVs. Moreover, we evaluated the strength of each SNP using the F statistic [19] (F = beta2/se2), and excluded SNPs with F < 10, because F > 10 suggested sufficient strength to ensure the validity of the SNPs. The summary characteristics of the selected SNPs in the study were shown in the supplementary documents 1. The allelic effects of certain SNPs diverged from those documented on the Phenoscanner website, potentially attributable to variations in population sources across distinct databases. We selected the SNPs characteristics from the original GWAS data.

### Statistical analysis

In this study, as the flow chart shown in Fig. 1, we extracted SNPs from exposure data and outcome data. The inverse variance weighted (IVW) [20], the MR-Egger [21], Wald ratio, weighted median, weighted mode and simple mode methods were employed to assess the causal relationship between exposure and outcome. In addition, Phenoscanner website could examine the pleiotropic effects of IVs, which is helping to remove confounding factors. We conducted Mendelian Randomization Pleiotropy Residual Sum and Outlier (MR-PRESSO) [22] test to identify the potential horizontal pleiotropic effects of the SNPs. *P* value of MR-PRESSO > 0.05 means the absence of horizontal pleiotropic effects. Heterogeneity test was performed using Cochran’s Q-test to identify whether the MR results were biased by the potential heterogenic factors. A leave-one-out permutation test was performed to assess whether the MR analysis results was biased by the influence of particular SNPs. And IVW was used for leave-one-out permutation test. All the analyses with *P* < 0.05 were considered statistically significant. All statistical analyses were performed using the R Studio (R version 4.2.3) software and the R package “TwoSampleMR”.

## Results

**Fig. 2:**
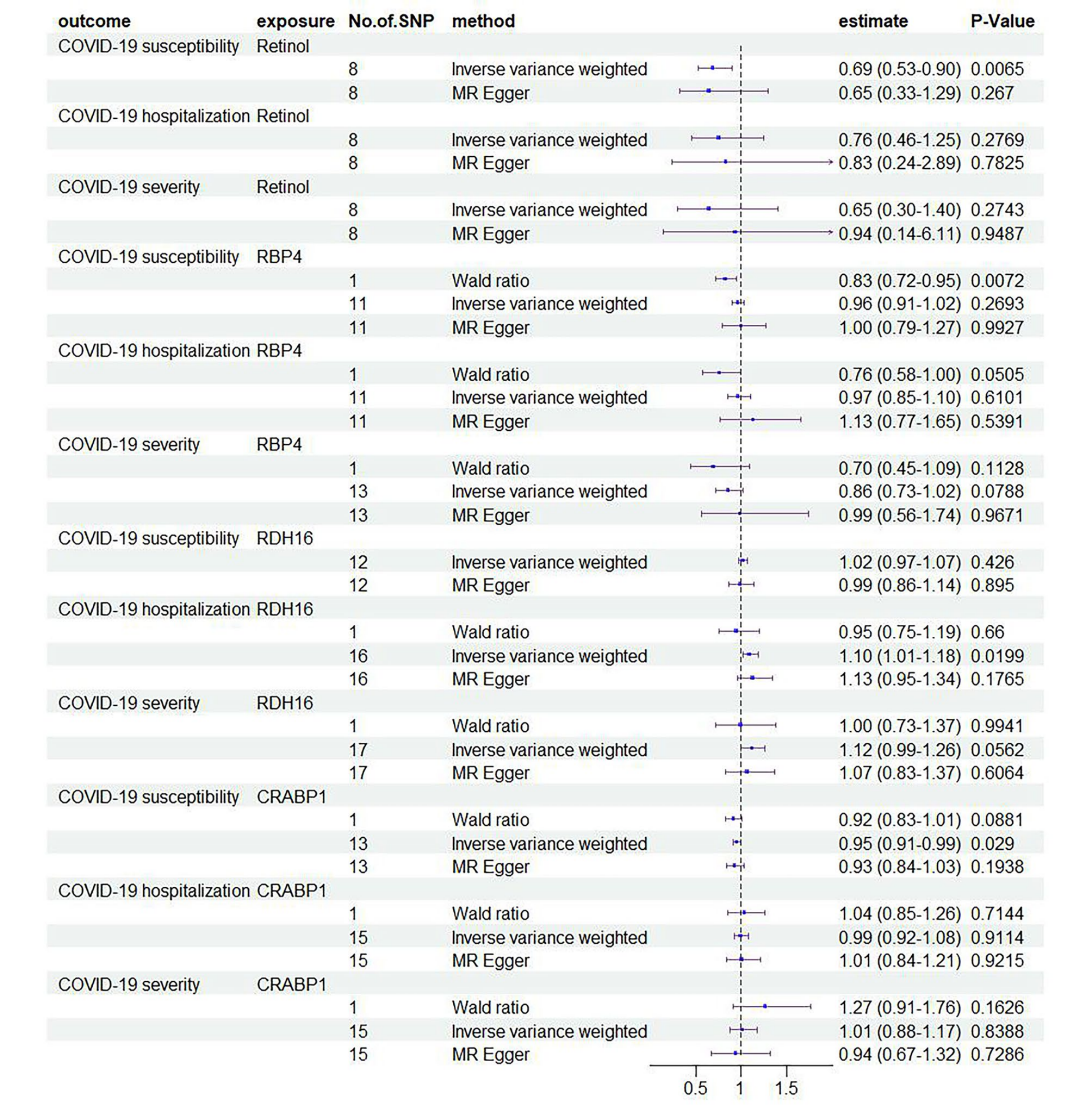
The forest plot for the causal effect of retinol and associated proteins on COVID-19 by MR analytical methods

The MR analyses using different methods including IVW, MR-PRESSO and Wald ratio, to estimate the causal inference of retinol, RBP4, RDH16 and CRABP1 on COVID-19 susceptibility and severity were presented in Fig. 2 and Table ​1. The MR results and leave-one-out analysis can be found in supplementary documents 2. In MR analysis between RBP4 and COVID-19 hospitalization, rs112357560 was deleted due to horizontal pleiotropy in MR-PRESSO. Horizontal pleiotropy in MR-PRESSO showed IVs could not influence outcomes directly through exposure factors, which violated the assumptions of MR. The F statistics which evaluated the strength of each SNP ranged from 20.80 to 87.83 for all the instrument SNPs, indicating an absence of weak instruments [23]. *P* < 0.05 were considered statistically significant.

Retinol reported a negative association with COVID-19 susceptibility (OR: 0.69, 95% CI: 0.53–0.90, P: 0.0065) using IVW, whereas the associations with the COVID-19 hospitalization or severity were insignificant. RBP4 was associated with COVID-19 susceptibility using the Wald ratio (OR: 0.83, 95% CI: 0.72–0.95, P: 0.0072) at a significance threshold (*P* < 5 × 10^− 8^), but the association was not significant (OR: 0.96, 95% CI: 0.91–1.02, P: 0.2693) at a liberal significance threshold (*P* < 5 × 10^− 6^). The associations between RBP4 with COVID-19 hospitalization (OR: 0.76, 95% CI: 0.58-1.00, P: 0.0505) and severity (OR: 0.86, 95% CI: 0.73–1.02, P: 0.0788) were not significant. IVW analysis showed a positive causal association between RDH16 and COVID-19 hospitalization (OR: 1.10, 95% CI: 1.01–1.18, P: 0.0199) using a liberal selection of genetic variants (*P* < 5 × 10^− 6^). However, no causal effect of RDH16 on COVID-19 severity (OR: 1.12, 95% CI: 0.99–1.26, P: 0.0562) was detected. CRABP1 was association with COVID-19 susceptibility (OR: 0.95, 95% CI: 0.91–0.99, P: 0.0290) using the IVW with a significance threshold (*P* < 5 × 10^− 6^). Other four MR methods also reported similar results. MR-PRESSO global test and the MR-Egger intercept test did not detect horizontal pleiotropy and Cochran Q tests showed no evidence for heterogeneity (Table 1).

**Table 1:** Mendelian randomization estimates for genetically predicted retinol and related proteins on different severities of COVID-19 using different methods with liberal instrument selection

Exposure	Outcome	P for significance threshold	MR methods	No. of SNPs	OR (95% CI)	MR P value	P-het	P-intercept	P for MR-PRESSO
Retinol	COVID-19 susceptibility	5 × 10^− 6^	IVW	8	**0.69 (0.53–0.90)**	0.0065	0.87	0.86	0.864
	COVID-19 susceptibility	5 × 10^− 6^	MR-Egger	8	0.65 (0.33–1.29)	0.2670			
	COVID-19 hospitalization	5 × 10^− 6^	IVW	8	0.76 (0.46–1.25)	0.2769	0.73	0.88	0.728
	COVID-19 hospitalization	5 × 10^− 6^	MR-Egger	8	0.83 (0.24–2.89)	0.7825			
	COVID-19 severity	5 × 10^− 6^	IVW	8	0.65 (0.30–1.40)	0.2743	0.68	0.69	0.674
	COVID-19 severity	5 × 10^− 6^	MR-Egger	8	0.94 (0.14–6.11)	0.9487			
RBP4	COVID-19 susceptibility	5 × 10^− 8^	Wald ratio	1	**0.83 (0.72–0.95)**	0.0072	NA	NA	NA
	COVID-19 susceptibility	5 × 10^− 6^	IVW	11	0.96 (0.91–1.02)	0.2693	0.11	0.76	0.096
	COVID-19 susceptibility	5 × 10^− 6^	MR-Egger	11	1.00 (0.79–1.27)	0.9927			
	COVID-19 hospitalization	5 × 10^− 8^	Wald ratio	1	0.76 (0.58-1.00)	0.0505	NA	NA	NA
	COVID-19 hospitalization	5 × 10^− 6^	IVW	11	0.97 (0.85–1.10)	0.6101	0.06	0.41	0.062
	COVID-19 hospitalization	5 × 10^− 6^	MR-Egger	11	1.13 (0.77–1.65)	0.5391			
	COVID-19 severity	5 × 10^− 8^	Wald ratio	1	0.70 (0.45–1.09)	0.1128	NA	NA	NA
	COVID-19 severity	5 × 10^− 6^	IVW	13	0.86 (0.73–1.02)	0.0788	0.13	0.62	0.147
	COVID-19 severity	5 × 10^− 6^	MR-Egger	13	0.99 (0.56–1.74)	0.9671			
RDH16	COVID-19 susceptibility	5 × 10^− 6^	IVW	12	1.02 (0.97–1.07)	0.4260	0.84	0.68	0.843
	COVID-19 susceptibility	5 × 10^− 6^	MR-Egger	12	0.99 (0.86–1.14)	0.8950			
	COVID-19 hospitalization	5 × 10^− 8^	Wald ratio	1	0.95 (0.75–1.19)	0.6600	NA	NA	NA
	COVID-19 hospitalization	5 × 10^− 6^	IVW	16	**1.10 (1.01–1.18)**	0.0199	0.59	0.69	0.637
	COVID-19 hospitalization	5 × 10^− 6^	MR-Egger	16	1.13 (0.95–1.34)	0.1765			
	COVID-19 severity	5 × 10^− 8^	Wald ratio	1	1.00 (0.73–1.37)	0.9941	NA	NA	NA
	COVID-19 severity	5 × 10^− 6^	IVW	17	1.12 (0.99–1.26)	0.0562	0.73	0.68	0.209
	COVID-19 severity	5 × 10^− 6^	MR-Egger	17	1.07 (0.83–1.37)	0.6064			
CRABP1	COVID-19 susceptibility	5 × 10^− 8^	Wald ratio	1	0.92 (0.83–1.01)	0.0881	NA	NA	NA
	COVID-19 susceptibility	5 × 10^− 6^	IVW	13	**0.95 (0.91–0.99)**	0.0290	0.71	0.60	0.341
	COVID-19 susceptibility	5 × 10^− 6^	MR-Egger	13	0.93 (0.84–1.03)	0.1938			
	COVID-19 hospitalization	5 × 10^− 8^	Wald ratio	1	1.04 (0.85–1.26)	0.7144	NA	NA	NA
	COVID-19 hospitalization	5 × 10^− 6^	IVW	15	0.99 (0.92–1.08)	0.9114	0.62	0.87	0.657
	COVID-19 hospitalization	5 × 10^− 6^	MR-Egger	15	1.01 (0.84–1.21)	0.9215			
	COVID-19 severity	5 × 10^− 8^	Wald ratio	1	1.27 (0.91–1.76)	0.1626	NA	NA	NA
	COVID-19 severity	5 × 10^− 6^	IVW	15	1.01 (0.88–1.17)	0.8388	0.20	0.63	0.263
	COVID-19 severity	5 × 10^− 6^	MR-Egger	15	0.94 (0.67–1.32)	0.7286			

Abbreviations: CI, confidence interval; CRABP1, Cellular retinoic acid-binding protein 1; IVW, inverse variance weighted; MR-PRESSO, Mendelian randomization-pleiotropy residual sum outlier; NA, not available; No. of SNPs, number of single nucleotide polymorphisms; OR, odds ratio; P-het, *p*-value for heterogeneity using Cochran Q test; P-intercept, *p*-value for MR-Egger intercept; RBP4, Retinol-binding protein 4; RDH16, Retinol dehydrogenase 16.

## Discussion

Previous studies had shown that VA plasma levels were reduced in COVID-19 patients with acute inflammation and severely reduced VA plasma levels were significantly associated with acute respiratory distress syndrome and mortality [24]. In the present study, based on the available GWAS, we investigate the causal effect of COVID-19 and retinol, RBP4, RDH16 and CRABP1 through two-sample MR approach. These results suggest causal associations between retinol and COVID-19 susceptibility, RBP4 and COVID-19 susceptibility, RDH16 and COVID-19 hospitalization, and CRABP1 and COVID-19 susceptibility.

SARS-CoV2 binding to angiotensin-converting enzyme 2 (ACE2) mediates cell entry. Then it triggers antiviral innate immune responses first. Viral RNA in the cytoplasm is recognized by RA-inducible gene-I (RIG-I) like receptor family proteins. RIG-I plays a major role in this progress and is responsible for type I interferon synthesis [25]. The expression and activity of RIG-I are enhanced by RA binding to the DNA [26]. With prolonged COVID-19 stimulation, retinol resources are used up [27]. The RIG-I pathway is deactivated after RA is depleted [28]. In addition, based on the fact that vitamin-like A enhances IFN-I and antiviral effects by activating RIG-I, RA has been proposed for inclusion in COVID-19 treatment regimens, especially in combination with type 1 interferon [29]. It has been suggested that VA binding to fatty acid binding sites in SARS-CoV-2 spike proteins as ligand may stabilize the blocked spike conformation and inhibit viral entry particularly early in the infection process [30]. This MR study also found that retinol is negatively associated with COVID-19 susceptibility, which is helpful in the research of COVID-19 prevention.

As previously mentioned, the production of multiple VA species such as all-trans RA requires RBP4 and various other proteins. RBP4 and VA levels were significantly decreased in patients hospitalized with COVID-19 during the acute phase of infection compared to patients recovering after a mild course of the disease. The reduced levels of RBP4 and VA possessed a significant correlation [10]. RBP4, the major transport protein of retinol in circulation, delivers retinol to tissues via binding to specific membrane receptors [31]. The release of retinol bound to RBP4 is under homeostatic control. During inflammation, the acute protein response boosts the production of hepatic inflammatory cytokines while simultaneously decreasing RBP4 release. Circulating VA decreases as a result of the subsequent reduction in holo-RBP4 (retinol bound to apo-RBP4) [32]. This MR study also found that the inverse correlation of RBP4 with COVID-19 susceptibility, which may indirectly support a causal link between VA and COVID-19.

A positive association is observed between RDH16 and COVID-19 hospitalization. This is contradictory to the role of RDH16 in RA signaling pathways. However, a study concluded that androgen dihydrotestosterone (DHT) increased endothelial injury mediated by SARS-CoV-2 spike protein [33]. Besides, RDH16 can oxidize the 3α-hydroxysteroids androstane-diol to DHT [34]. In our MR study, the positive association between RDH16 with COVID-19 hospitalization were also found, which requires further confirmation by future research.

In the genomic RA signaling pathway, RA leads to the transcription of various target genes. In non-genomic mechanisms, retinol binds to STRA6 and CRABP-1, activating cytokine signaling, such as Jak/STAT pathway, that influences the expression and signaling of cytokines and interferons [28]. Our study identified suggestive inverse associations for CRABP1 with COVID-19 susceptibility.

There remain limitations in our study. First of all, we used summary-level data, however, summarized data do not allow stratification by factors such as sex, age, adiposity, diet, and co-morbidities. Secondly, the number of SNPs was limited at the genome-wide significance threshold. Third, the GWAS data of RBP4, RDH16 and CRABP1 used to generate instruments were relatively small compared to retinol. Fourth, the exposure and outcome population of this study are European. The findings from the MR study based on European ancestry may not be applicable to other ethnic groups. Finally, leave-one-out analysis detected potential influential SNP for the above MR analyses, such as retinol on COVID-19 susceptibility, RDH16 on COVID-19 hospitalization, CRABP1 on COVID-19 susceptibility. We acknowledge the possibility that statistical assumptions may not have been met, thus additional tests or replication is needed.

## Conclusion

In conclusion, using large-scale genetic summary data, our study strengthens the evidence for a causal relationship between COVID-19 and RA signaling pathway related proteins. Our study defines that retinol is significantly associated with lower COVID-19 susceptibility, which provides a reference for the prevention of COVID-19 with vitamin A supplementation.

### Electronic supplementary material

Below is the link to the electronic supplementary material.

Supplementary Material 1

Supplementary Material 2

## Data Availability

All data generated or analysed during this study are included in this published article and its supplementary information files.
