# Supplementary material for: Retinol and retinol binding protein 4 levels and COVID-19: a Mendelian randomization study
Source: BMC Pulm Med. 2024 Apr 26;24:206. doi: 10.1186/s12890-024-03013-w (PMC11046857; doi:10.1186/s12890-024-03013-w)
Supplement: Supplementary file 1 — Supplementary Material 1 [file 12890_2024_3013_MOESM1_ESM.docx]

**Supplementary material 2**

**Retinol on COVID-19 susceptibility**

**SNP effect on Retinol || id: ukb-b-17406**

**SNP effect on COVID-19 susceptibility || id: ebi-a-GCST011072**


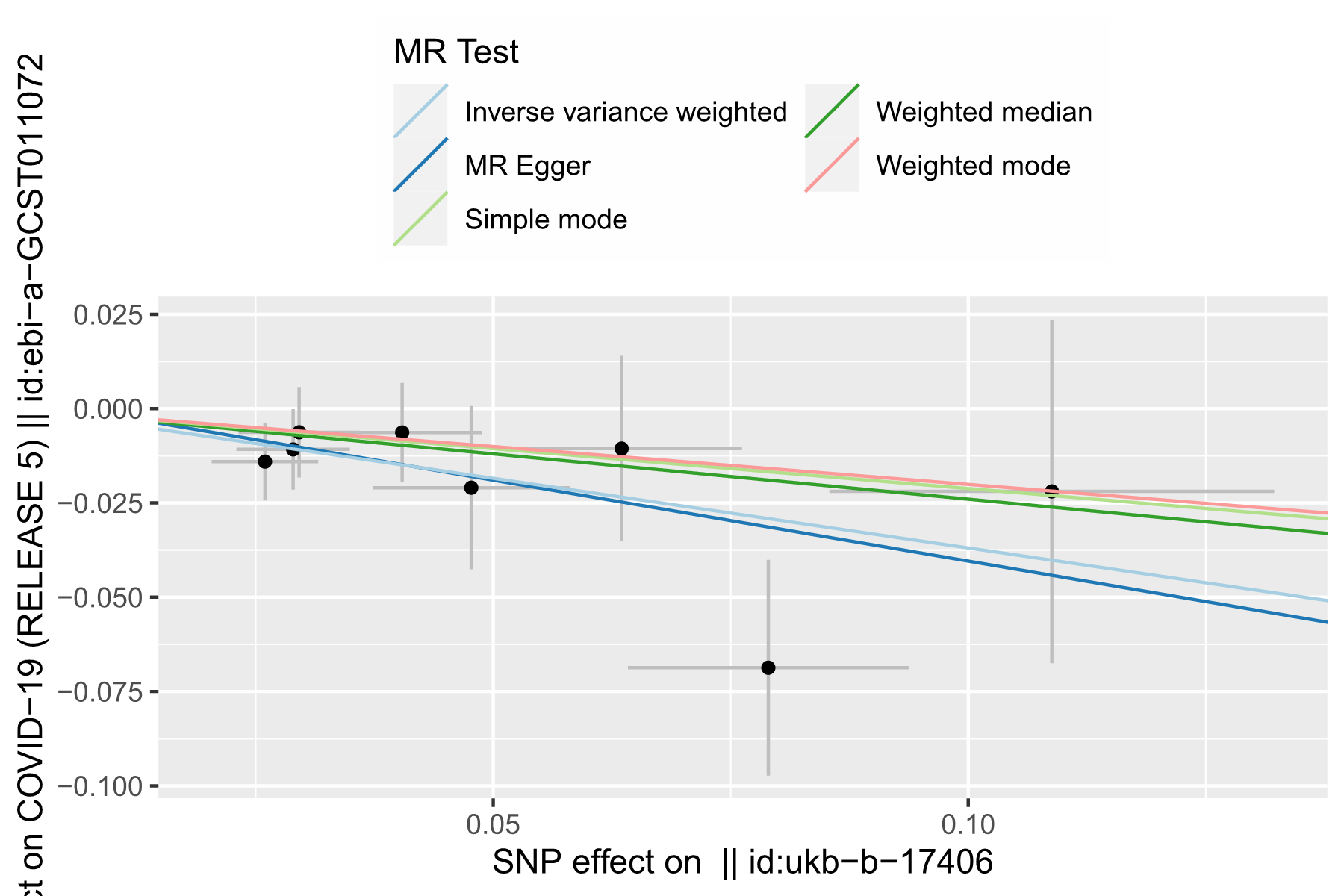


**Figure.S1 Scatter plot to visualize the causal effect of Retinol on COVID-19 susceptibility. The slope of the straight line indicates the magnitude of the causal association. IVW indicates inverse-variance weighted, and MR, Mendelian randomization.**


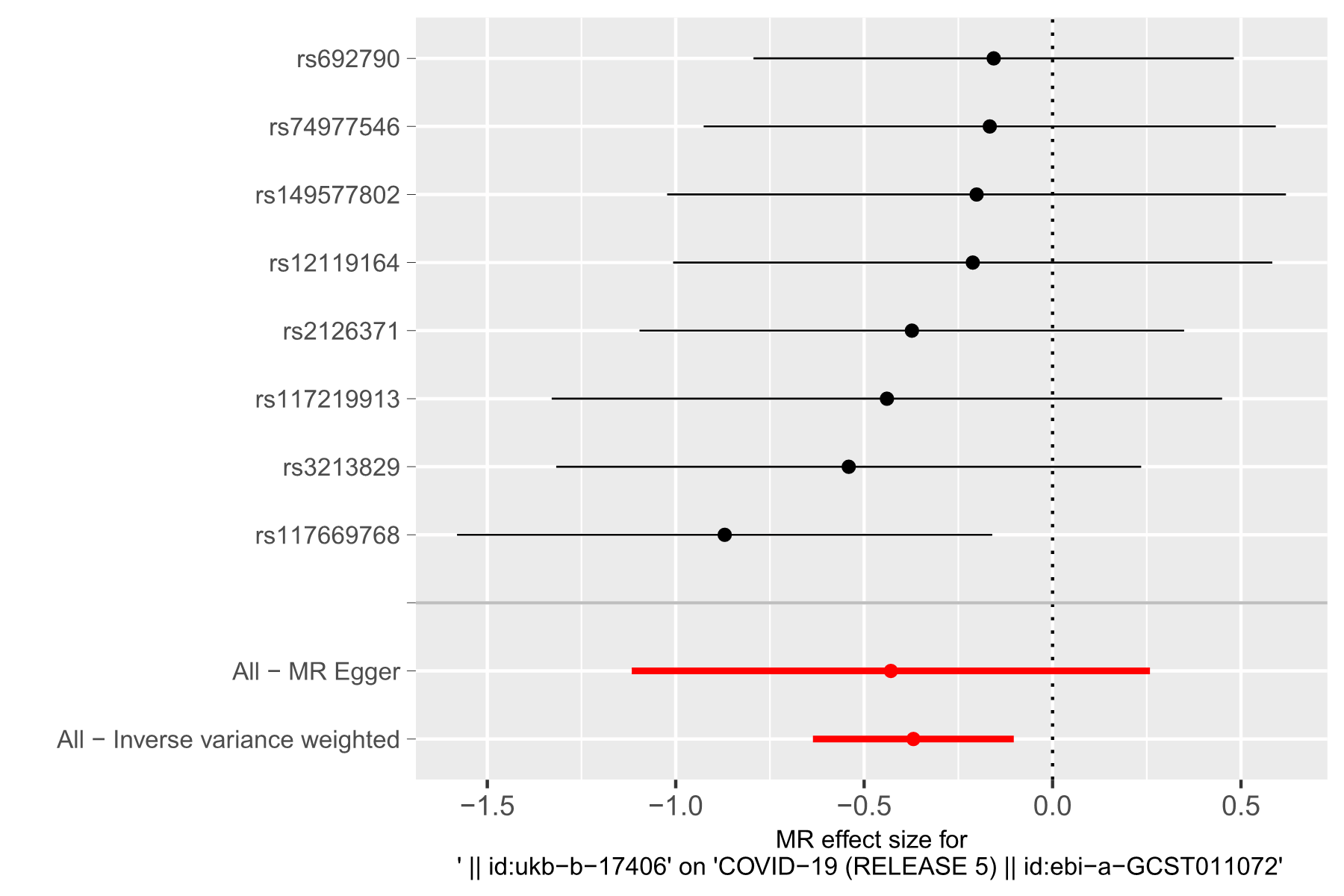


**MR effect size for Retinol || id: ukb-b-17406 on COVID-19 susceptibility || id: ebi-a-GCST011072**

**Figure.S2 Fixed-effect IVW analysis and of the causal association of Retinol on COVID-19 susceptibility. The black dots and bars indicated the causal estimate and 95% CI using each SNP. The red dot and bar indicated the overall estimate and 95% CI meta-analyzed by fixed-effect inverse variance weighted** **method and MR-Egger method.**


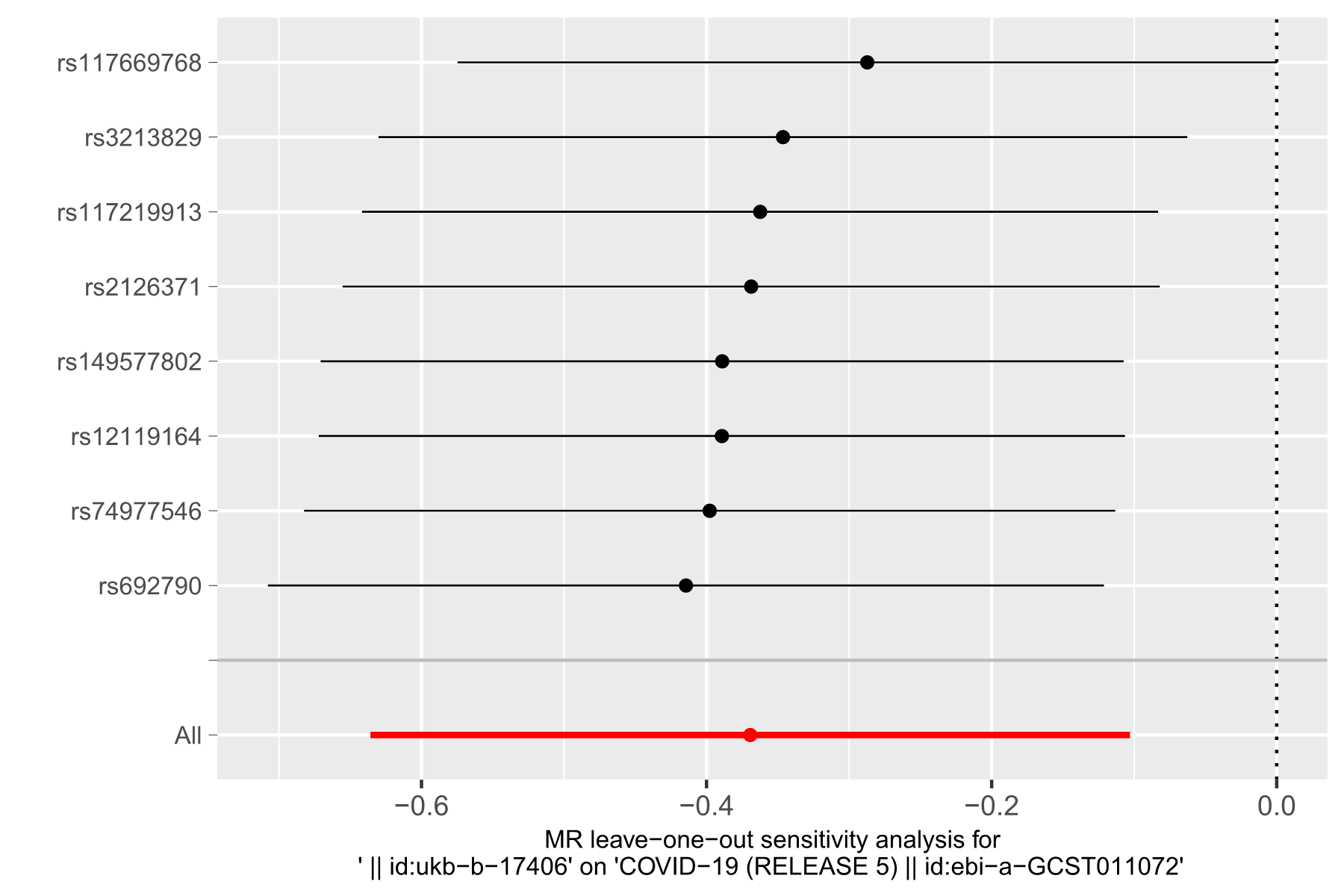


**MR leave-one-out sensitivity analysis for Retinol || id: ukb-b-17406 on COVID-19 susceptibility || id: ebi-a-GCST011072**

**Figure.S3 MR leave-one-out sensitivity analysis for** **Retinol on COVID-19 susceptibility. Circles indicate MR estimates for Retinol on COVID-19 susceptibility using inverse-variance weighted fixed-effect method if each single nucleotide polymorphism was omitted. The bars indicate the CI. MR indicates Mendelian randomization.**

**Retinol on COVID-19 hospitalization**

**SNP effect on COVID-19 hospitalization || id: ebi-a-GCST011081**


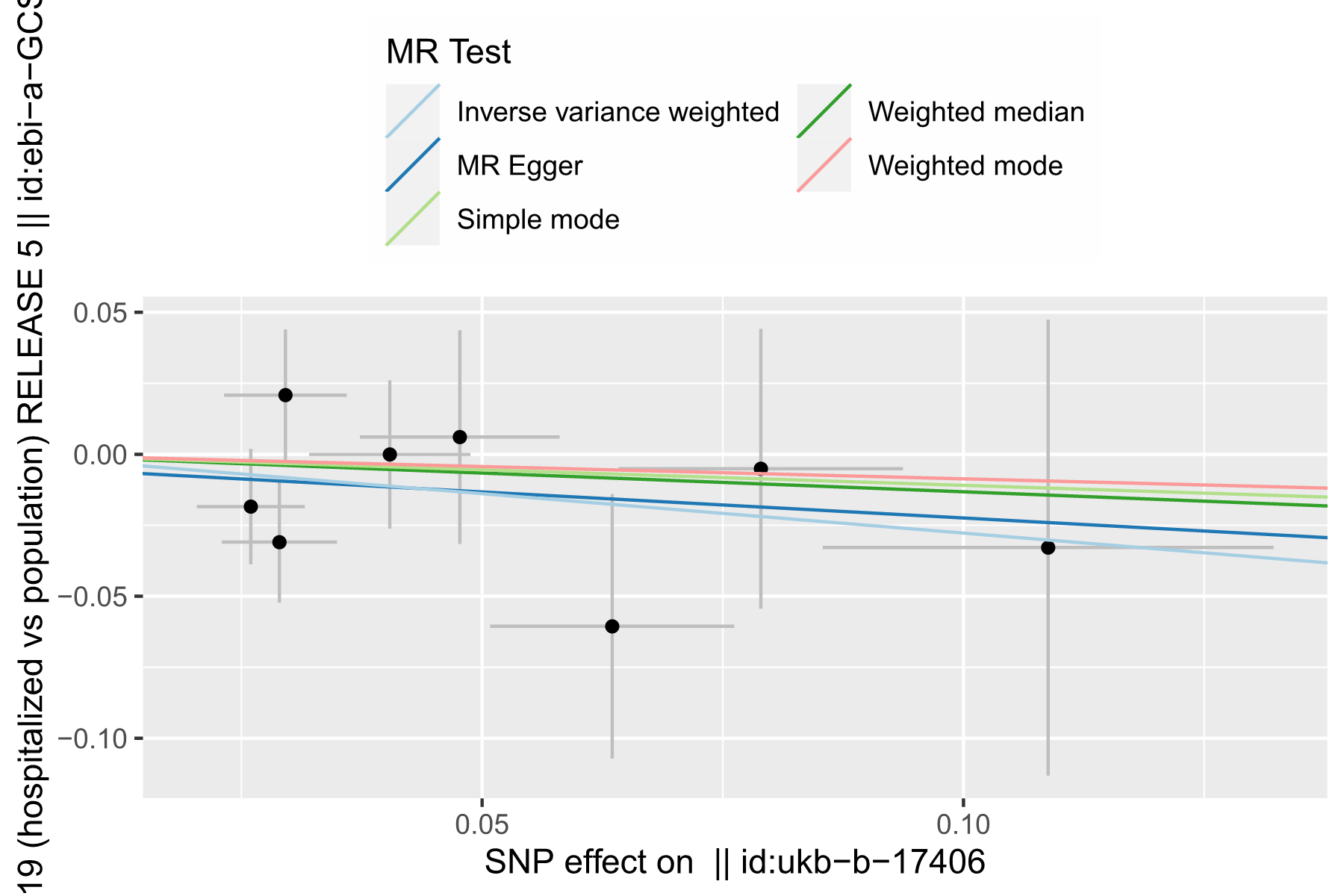


**SNP effect on Retinol || id: ukb-b-17406**

**Figure.S4 Scatter plot to visualize the causal effect of** **Retinol on COVID-19 hospitalization. The slope of the straight line indicates the magnitude of the causal association. IVW indicates inverse-variance weighted, and MR, Mendelian randomization.**


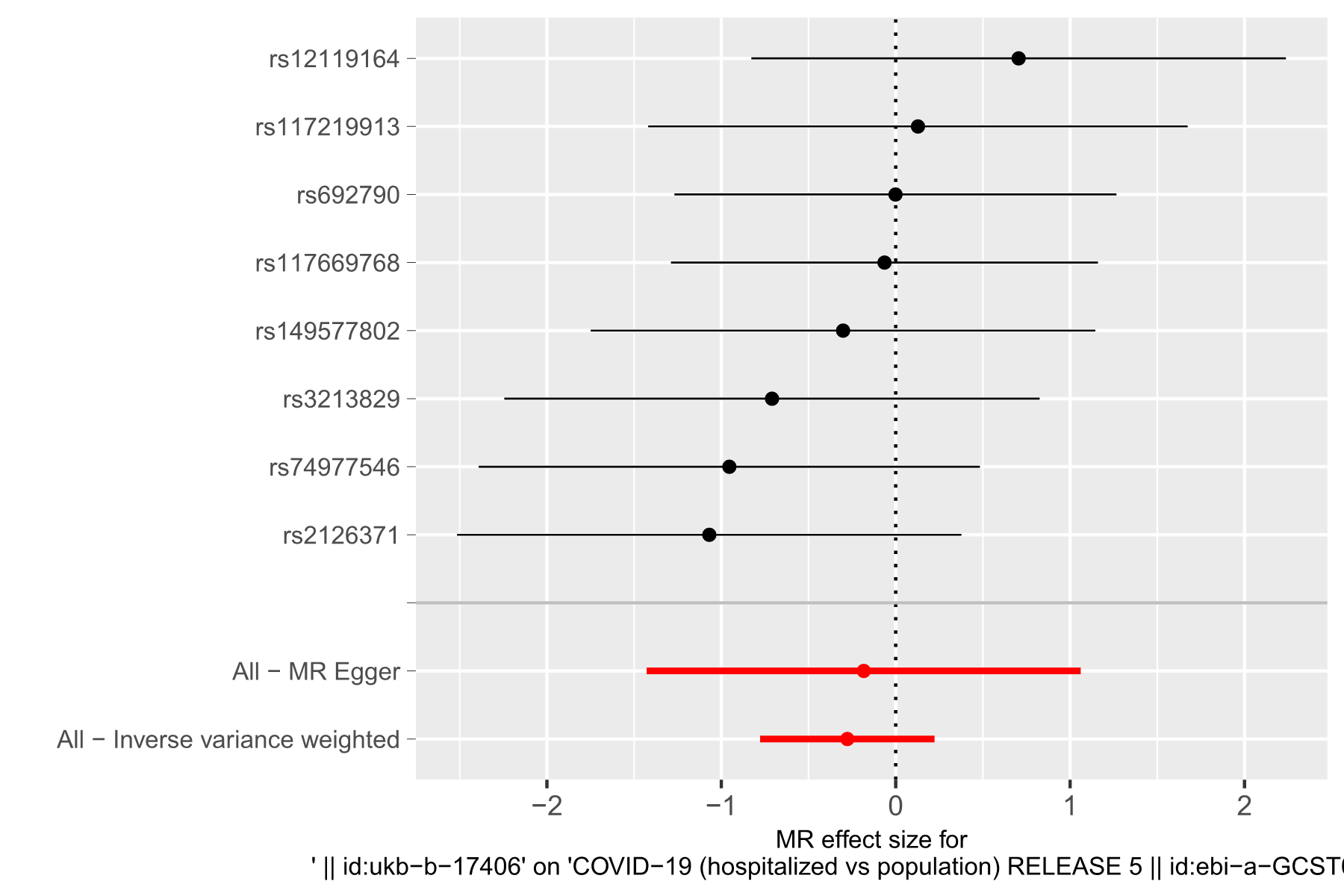


**MR effect size for Retinol || id: ukb-b-17406 on COVID-19 hospitalization || id: ebi-a-GCST011081**

**Figure.S5 Fixed-effect IVW analysis and of the causal association of Retinol on COVID-19 hospitalization. The black dots and bars indicated the causal estimate and 95% CI using each SNP. The red dot and bar indicated the overall estimate and 95% CI meta-analyzed by fixed-effect inverse variance weighted method and MR-Egger method.**


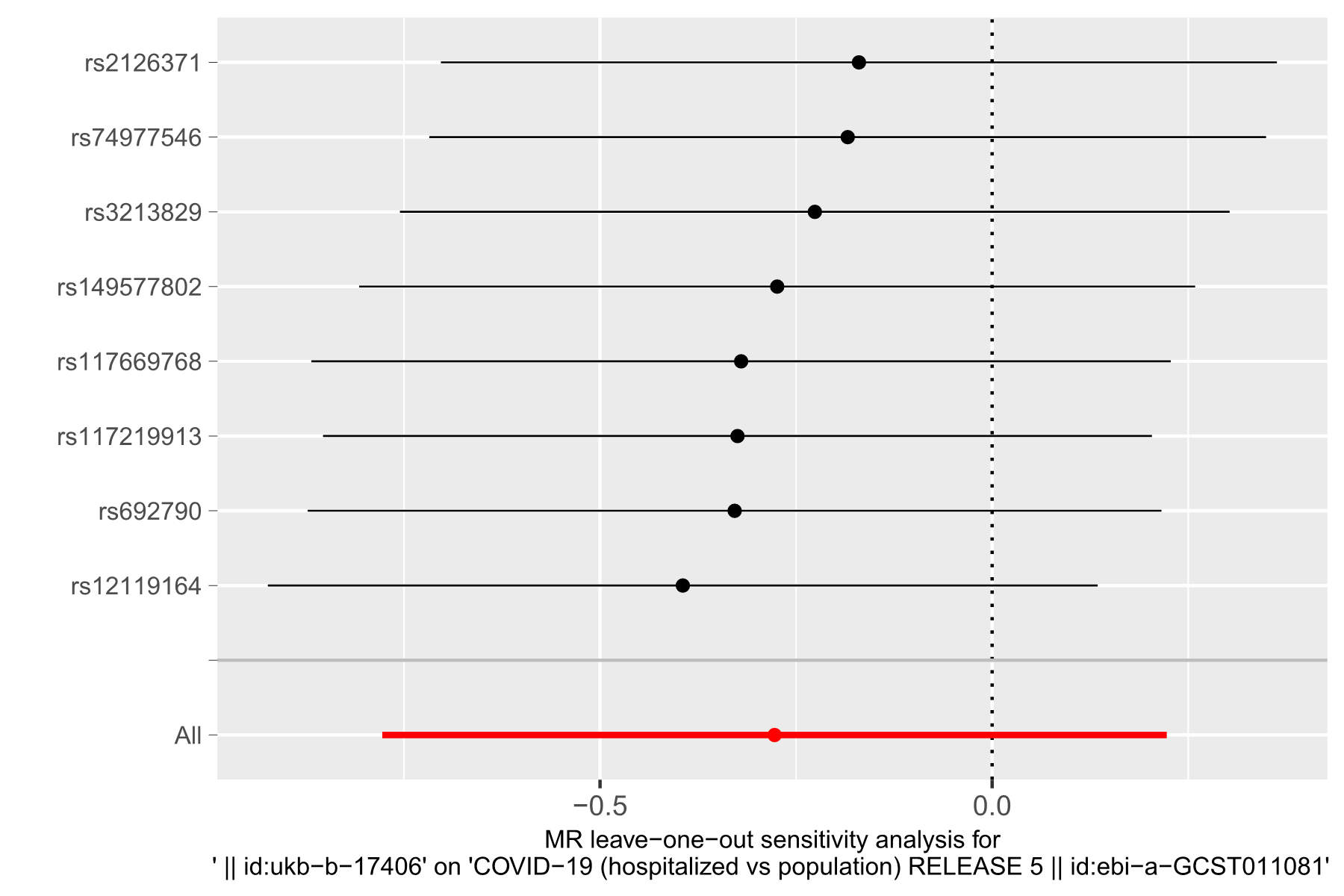


**MR leave-one-out sensitivity analysis for Retinol || id: ukb-b-17406 on COVID-19 hospitalization || id: ebi-a-GCST011081**

**Figure.S6 MR leave-one-out sensitivity analysis for** **Retinol on COVID-19 hospitalization. Circles indicate MR estimates for Retinol on** COVID-19 hospitalization **using inverse-variance weighted fixed-effect method if each single nucleotide polymorphism was omitted. The bars indicate the CI. MR indicates Mendelian randomization.**

**Retinol on COVID-19 severity**


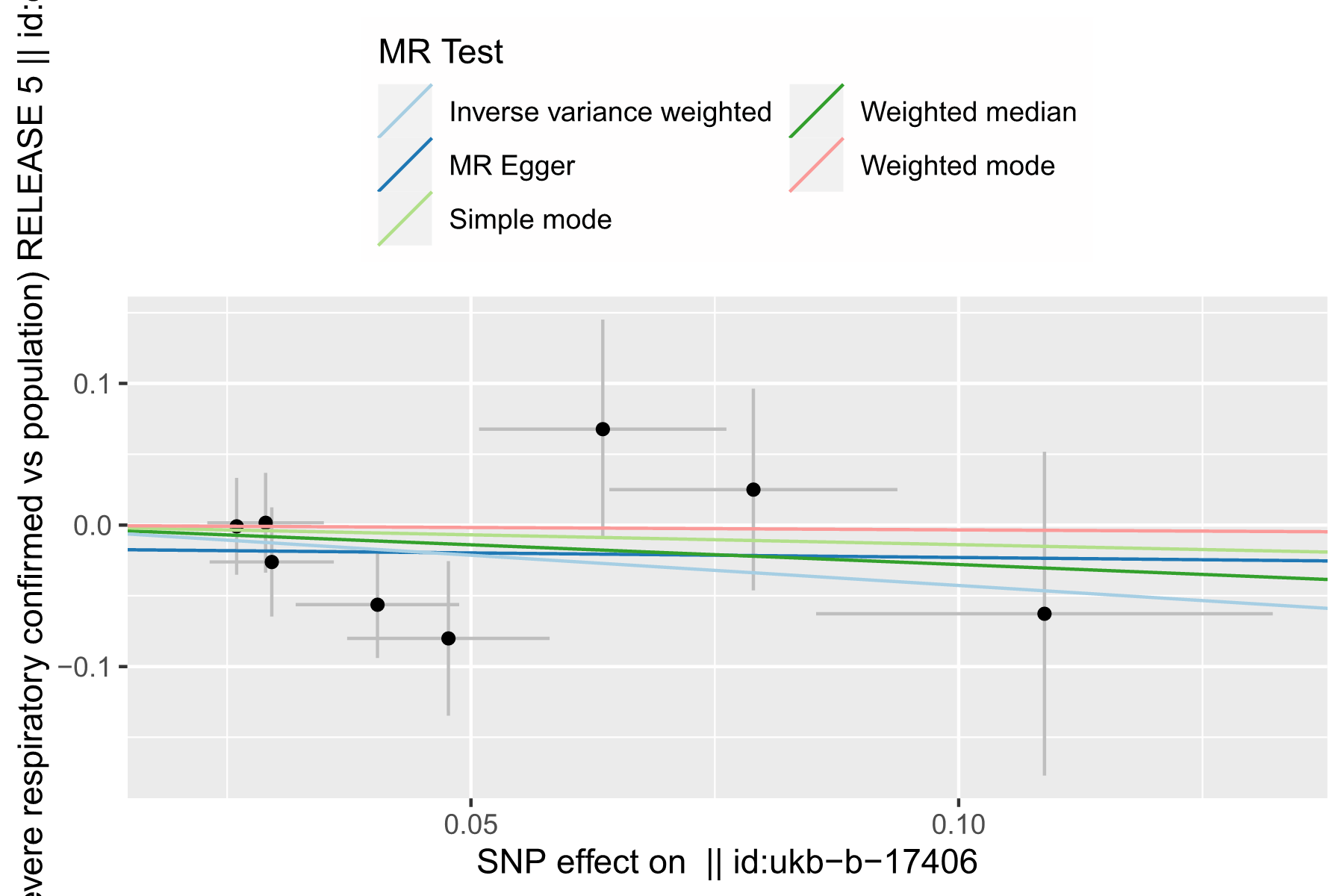


**SNP effect on COVID-19 severity || id: ebi-a-GCST011075**

**SNP effect on Retinol || id: ukb-b-17406**

**Figure.S7 Scatter plot to visualize the causal effect of Retinol on COVID-19 severity. The slope of the straight line indicates the magnitude of the causal association. IVW indicates inverse-variance weighted, and MR, Mendelian randomization.**


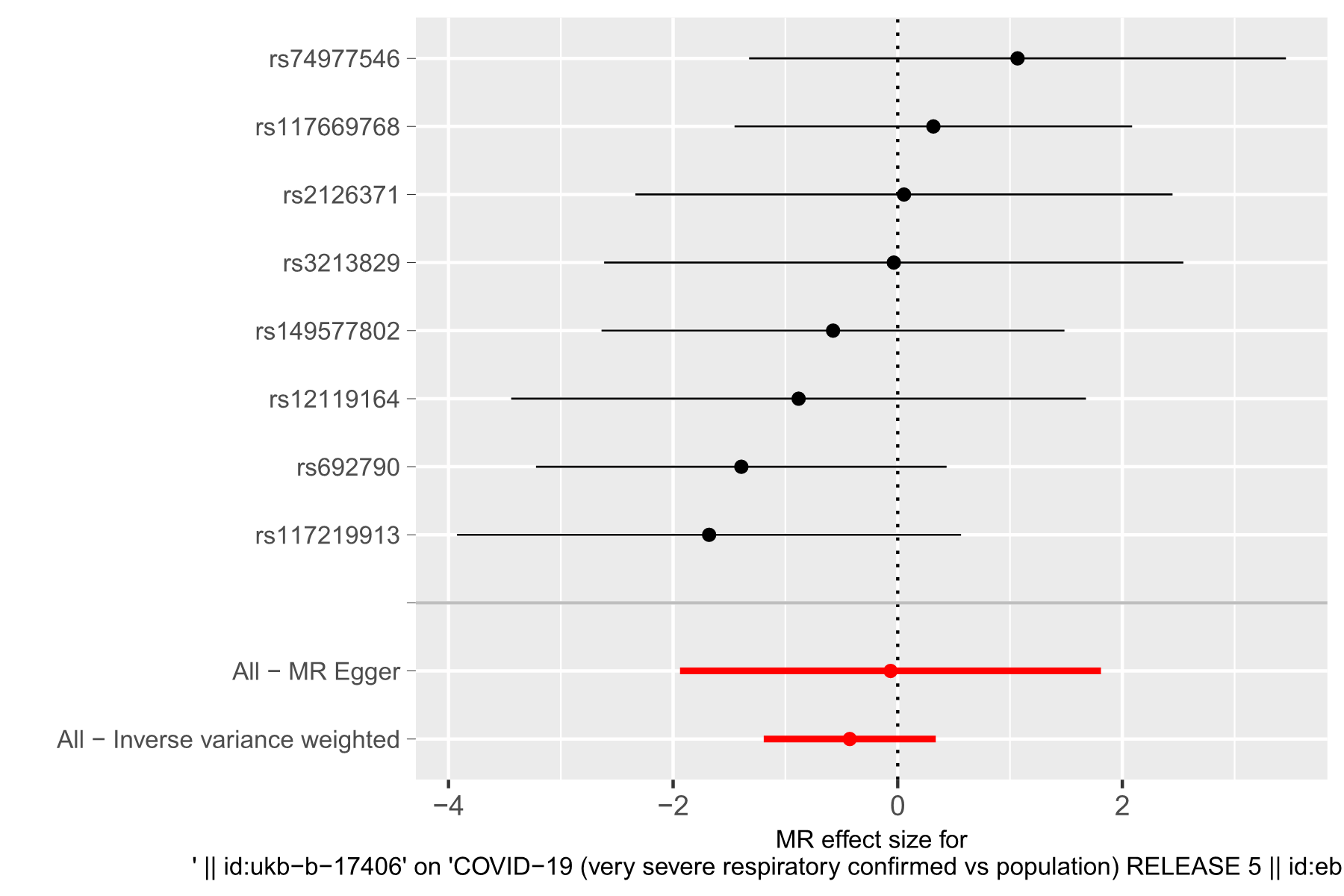


**MR effect size for Retinol || id: ukb-b-17406 on COVID-19 severity || id: ebi-a-GCST011075**

**Figure.S8 Fixed-effect IVW analysis and of the causal association of Retinol on COVID-19 severity. The black dots and bars indicated the causal estimate and 95% CI using each SNP. The red dot and bar indicated the overall estimate and 95% CI meta-analyzed by fixed-effect inverse variance weighted method and MR-Egger method.**


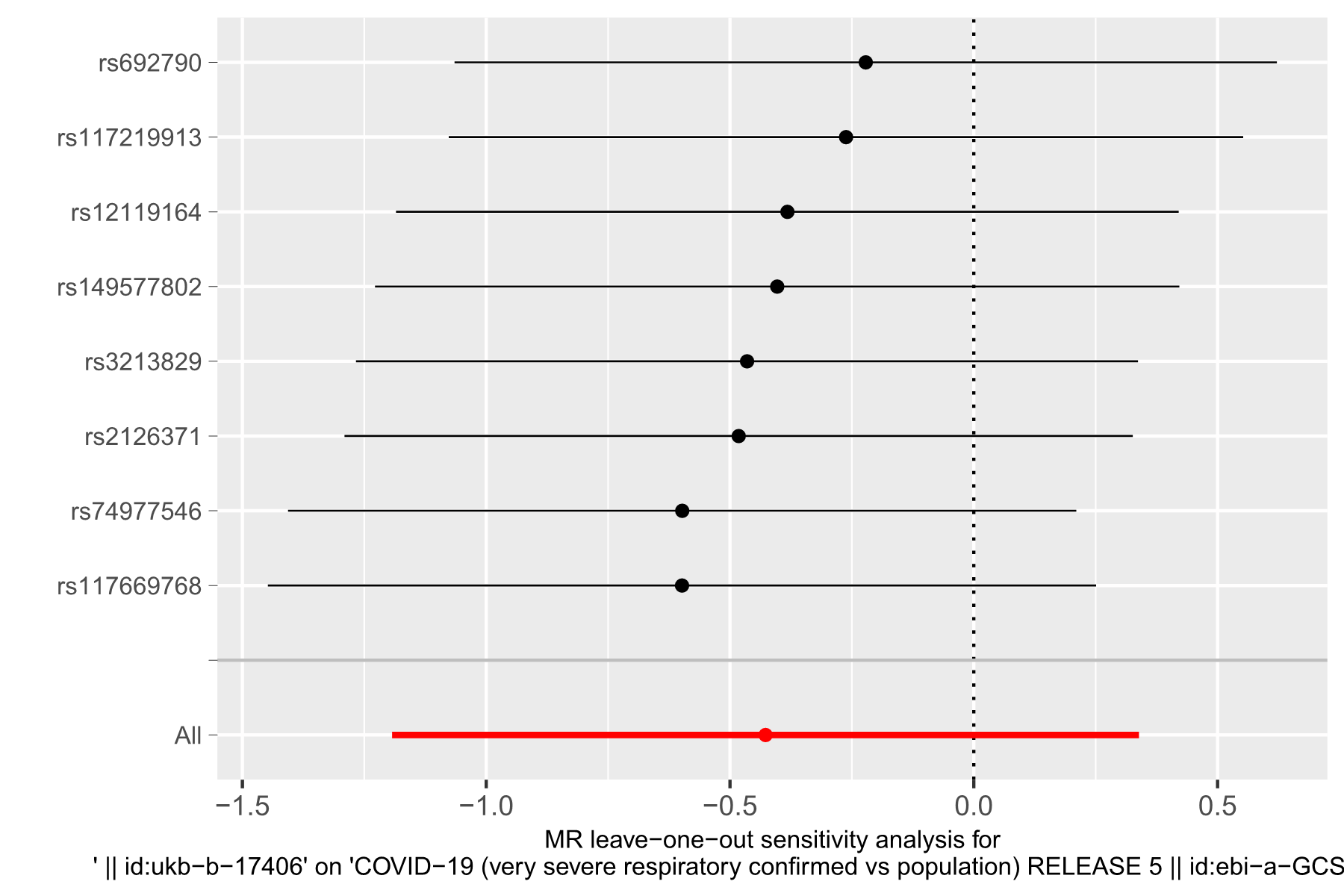


**MR leave-one-out sensitivity analysis for Retinol || id: ukb-b-17406 on COVID-19 severity || id: ebi-a-GCST011075**

**Figure.S9 MR leave-one-out sensitivity analysis for** **Retinol on COVID-19 severity. Circles indicate MR estimates for Retinol on** COVID-19 severity **using inverse-variance weighted fixed-effect method if each single nucleotide polymorphism was omitted. The bars indicate the CI. MR indicates Mendelian randomization.**

**RBP4 on COVID-19 susceptibility**


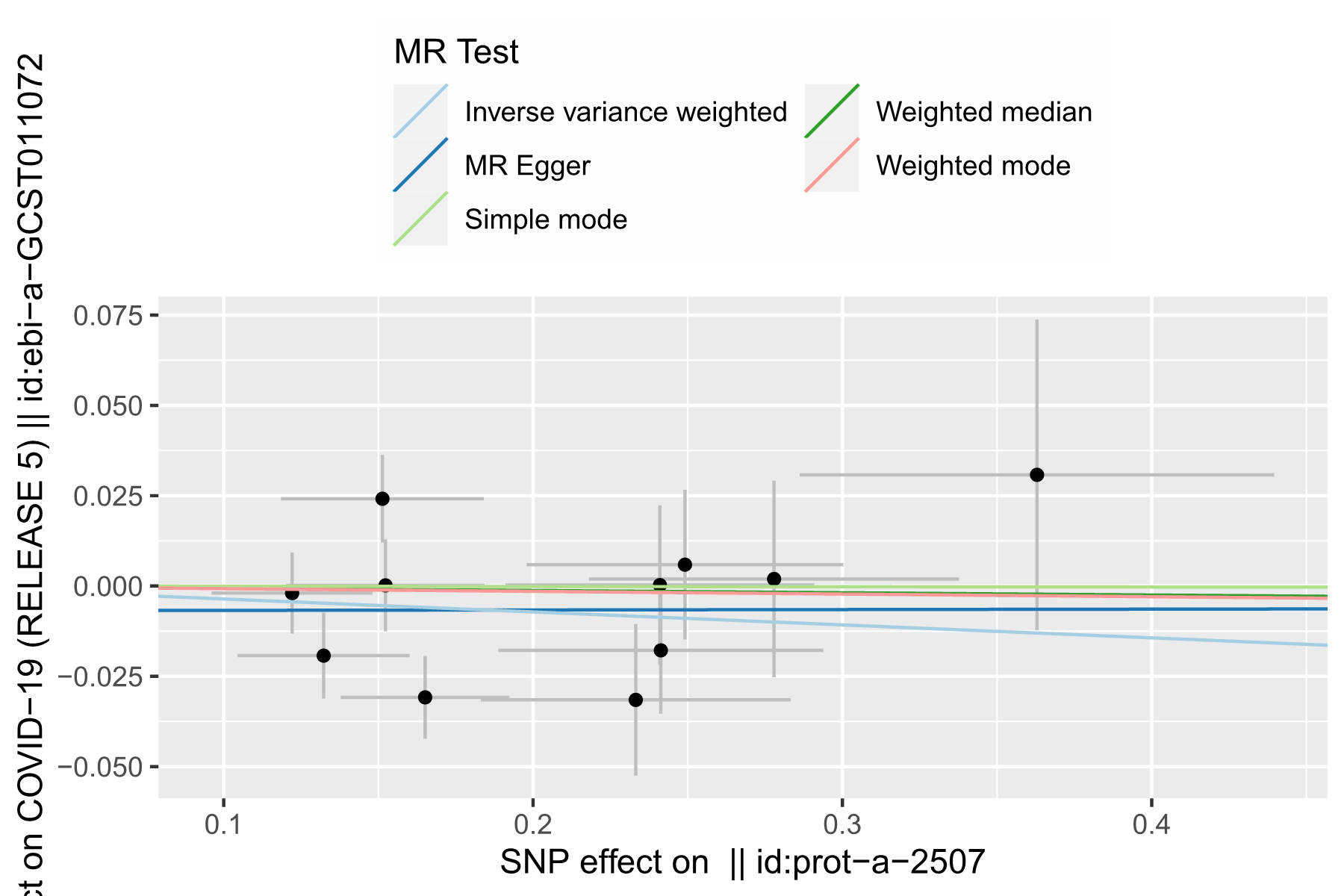


**SNP effect on COVID-19 susceptibility || id: ebi-a-GCST011072**

**SNP effect on RBP4 || id: prot-a-2507**

**Figure.S10 Scatter plot to visualize the causal effect of RBP4 on COVID-19 susceptibility. The slope of the straight line indicates the magnitude of the causal association. IVW indicates inverse-variance weighted, and MR, Mendelian randomization.**


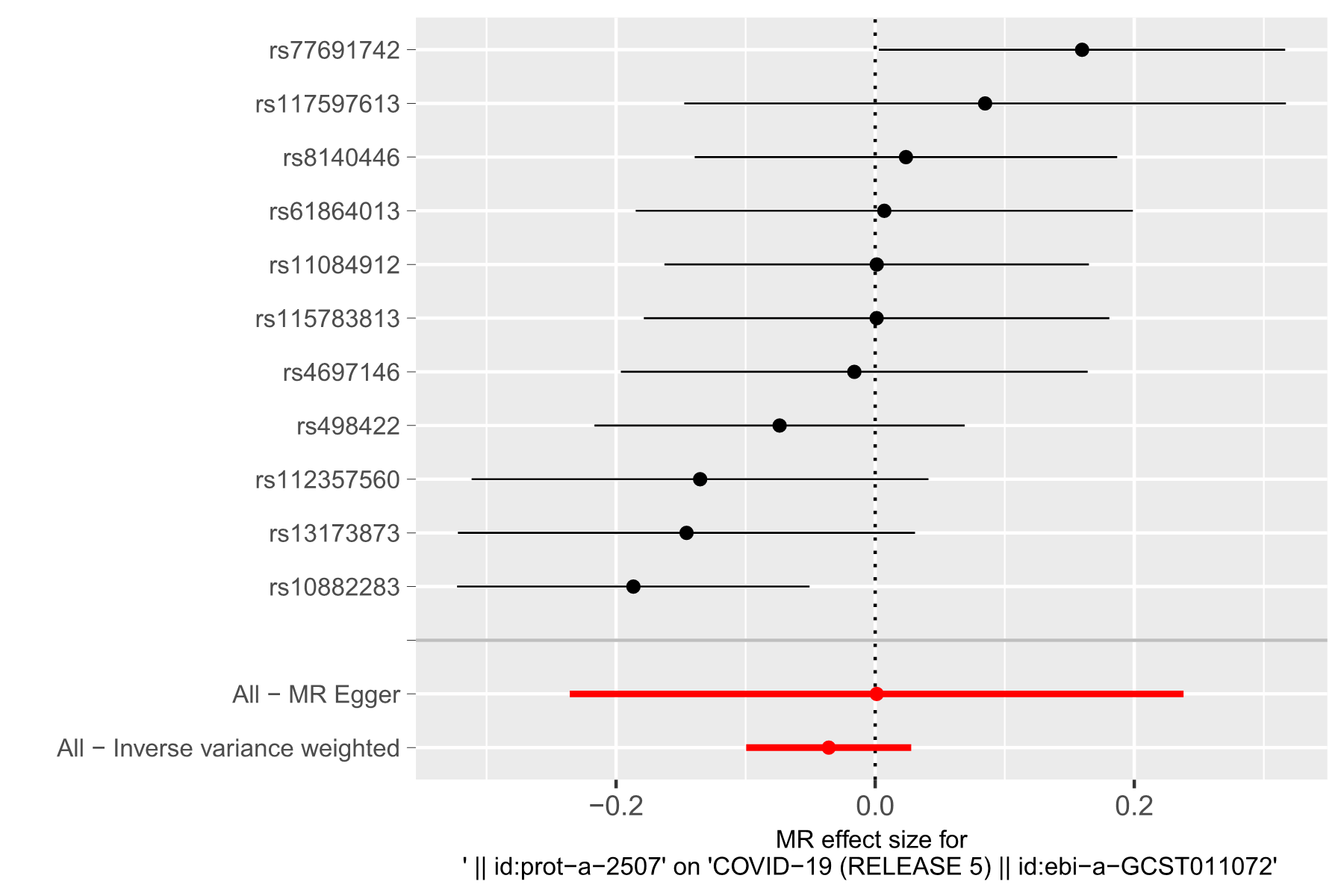


**MR effect size for RBP4 || id: prot-a-2507 on COVID-19 susceptibility || id: ebi-a-GCST011072**

**Figure.S11 Fixed-effect IVW analysis and of the causal association of RBP4 on COVID-19 susceptibility. The black dots and bars indicated the causal estimate and 95% CI using each SNP. The red dot and bar indicated the overall estimate and 95% CI meta-analyzed by fixed-effect inverse variance weighted method and MR-Egger method.**


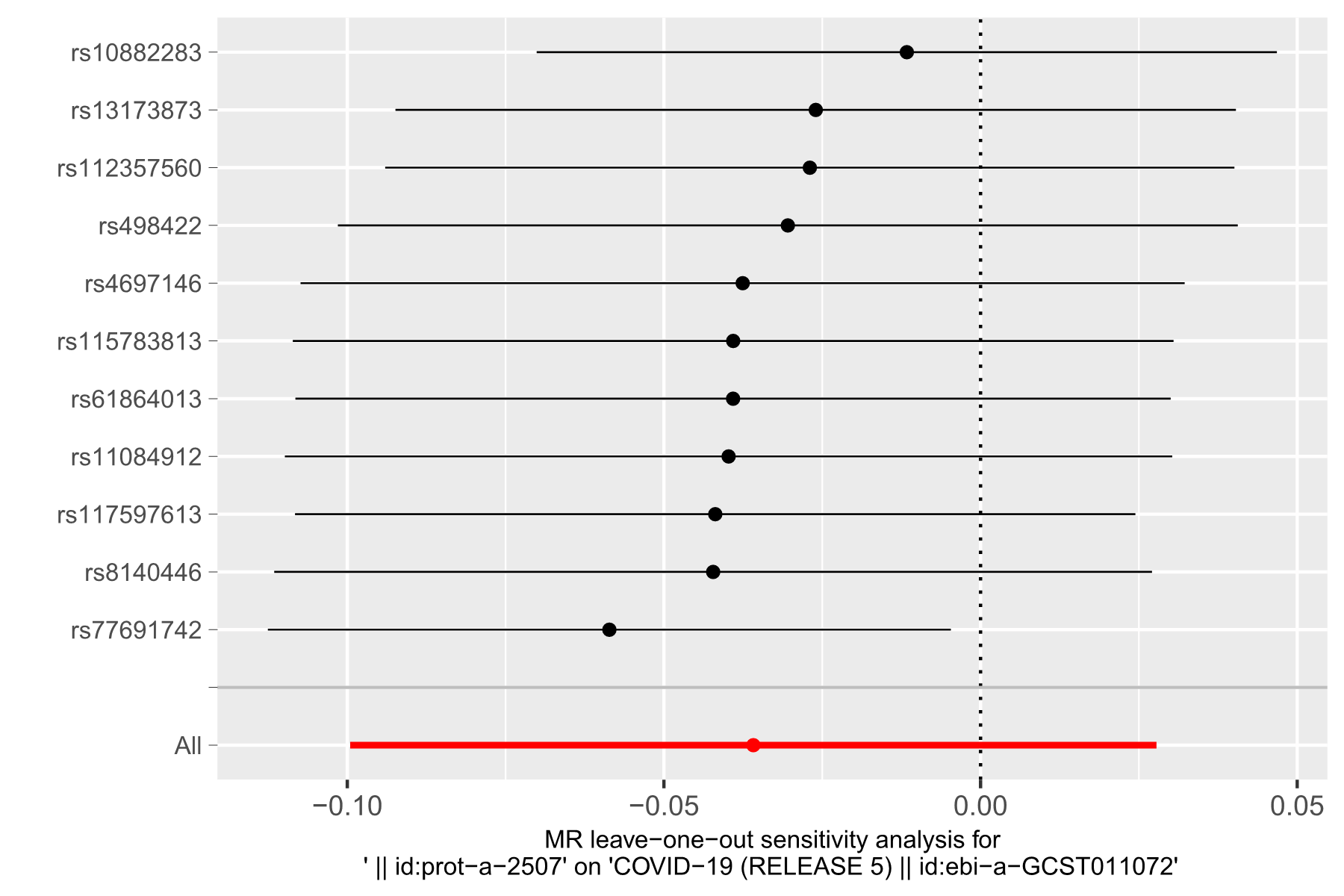


**MR leave-one-out sensitivity analysis for RBP4 || id: prot-a-2507 on COVID-19 susceptibility || id: ebi-a-GCST011072**

**Figure.S12 MR leave-one-out sensitivity analysis for** **RBP4 on COVID-19 susceptibility. Circles indicate MR estimates for RBP4 on** COVID-19 susceptibility **using inverse-variance weighted fixed-effect method if each single nucleotide polymorphism was omitted. The bars indicate the CI. MR indicates Mendelian randomization.**

**RBP4 on COVID-19 hospitalization**

**SNP effect on COVID-19 hospitalization || id: ebi-a-GCST011081**


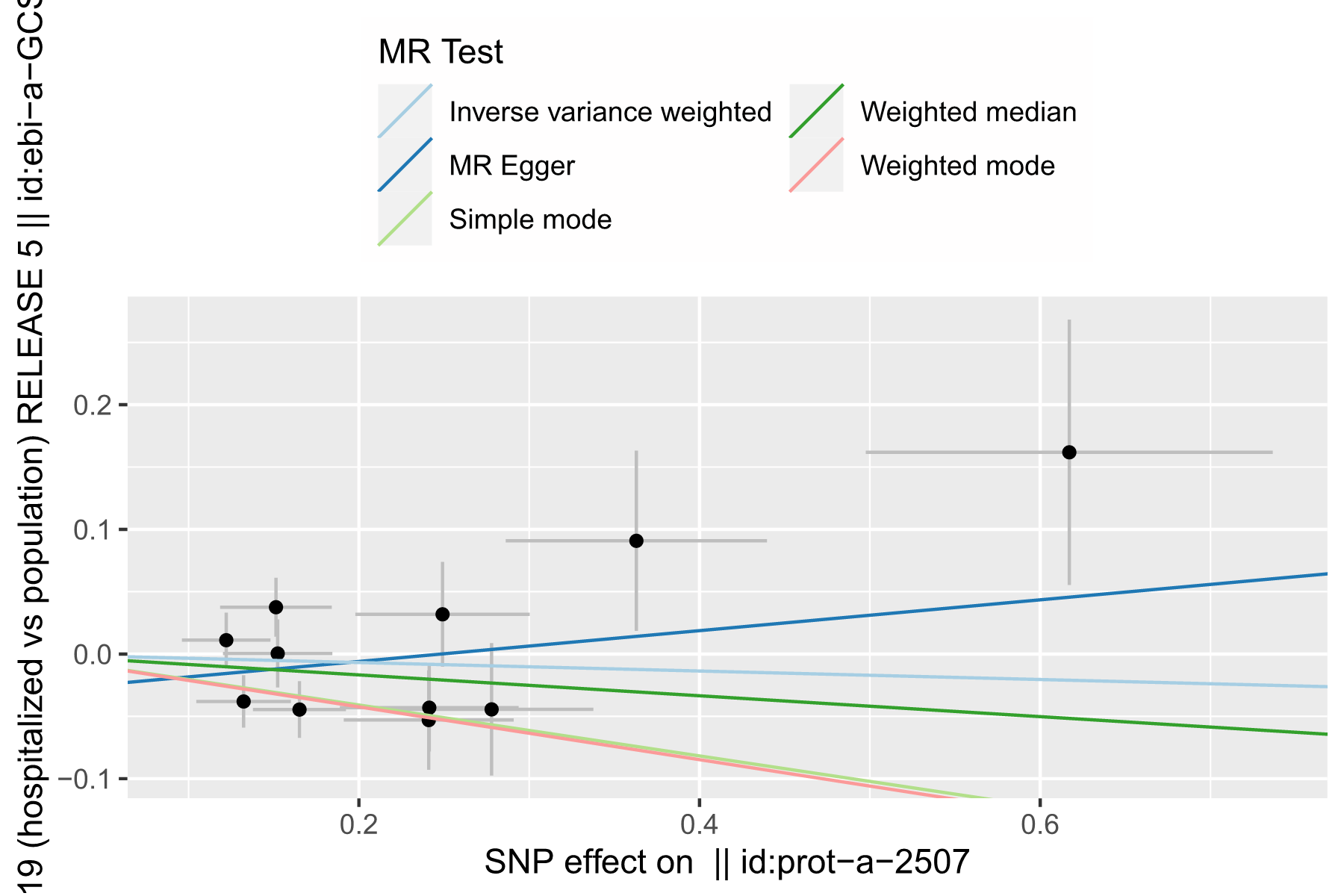


**SNP effect on RBP4 || id: prot-a-2507**

**Figure.S13** **Scatter plot to visualize the causal effect of RBP4 on COVID-19 hospitalization. The slope of the straight line indicates the magnitude of the causal association. IVW indicates inverse-variance weighted, and MR, Mendelian randomization.**


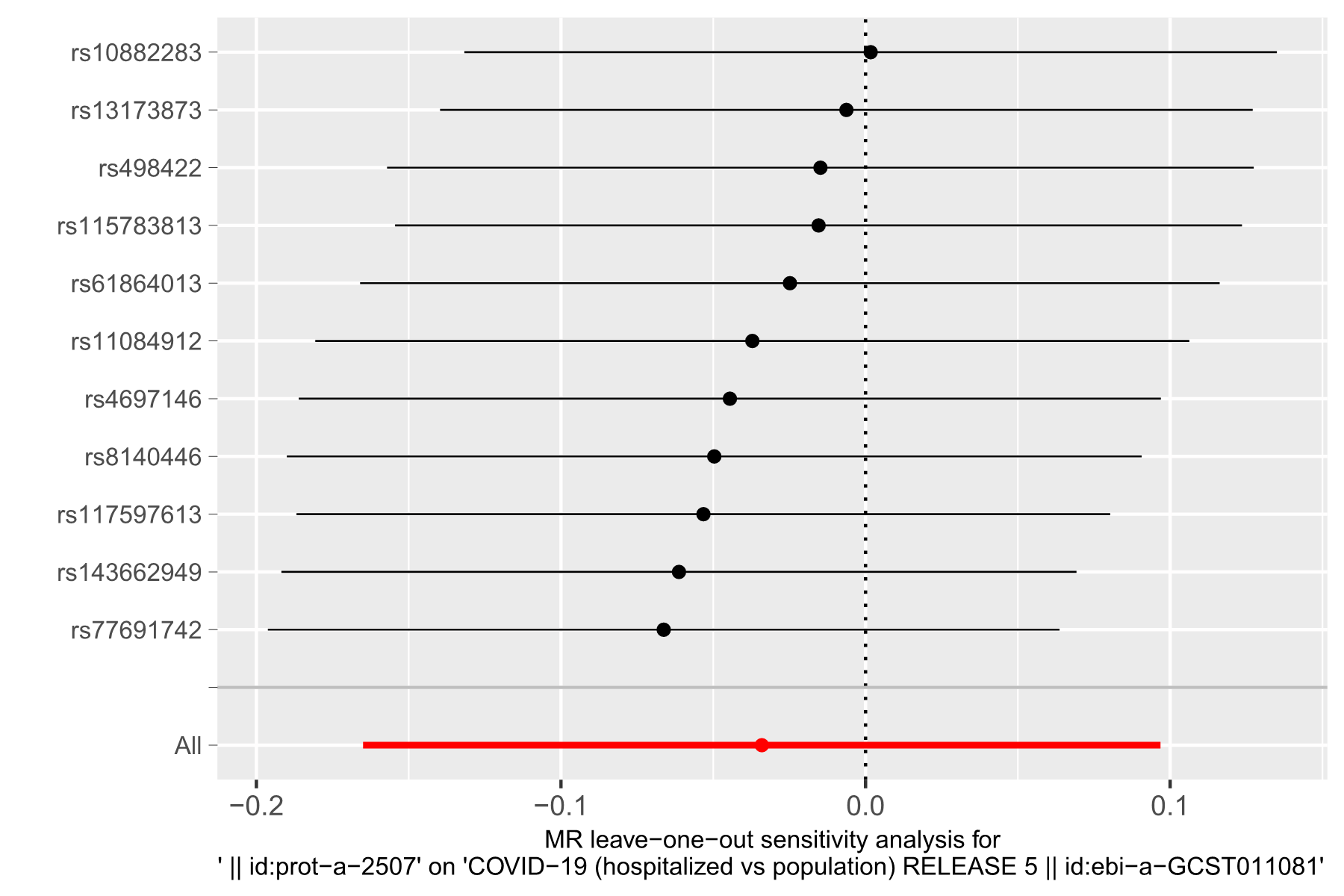


**MR effect size for RBP4 || id: prot-a-2507 on COVID-19 hospitalization || id: ebi-a-GCST011081**

**Figure.S14 Fixed-effect IVW analysis and of the causal association of RBP4 on COVID-19 hospitalization. The black dots and bars indicated the causal estimate and 95% CI using each SNP. The red dot and bar indicated the overall estimate and 95% CI meta-analyzed by fixed-effect inverse variance weighted method and MR-Egger method.**


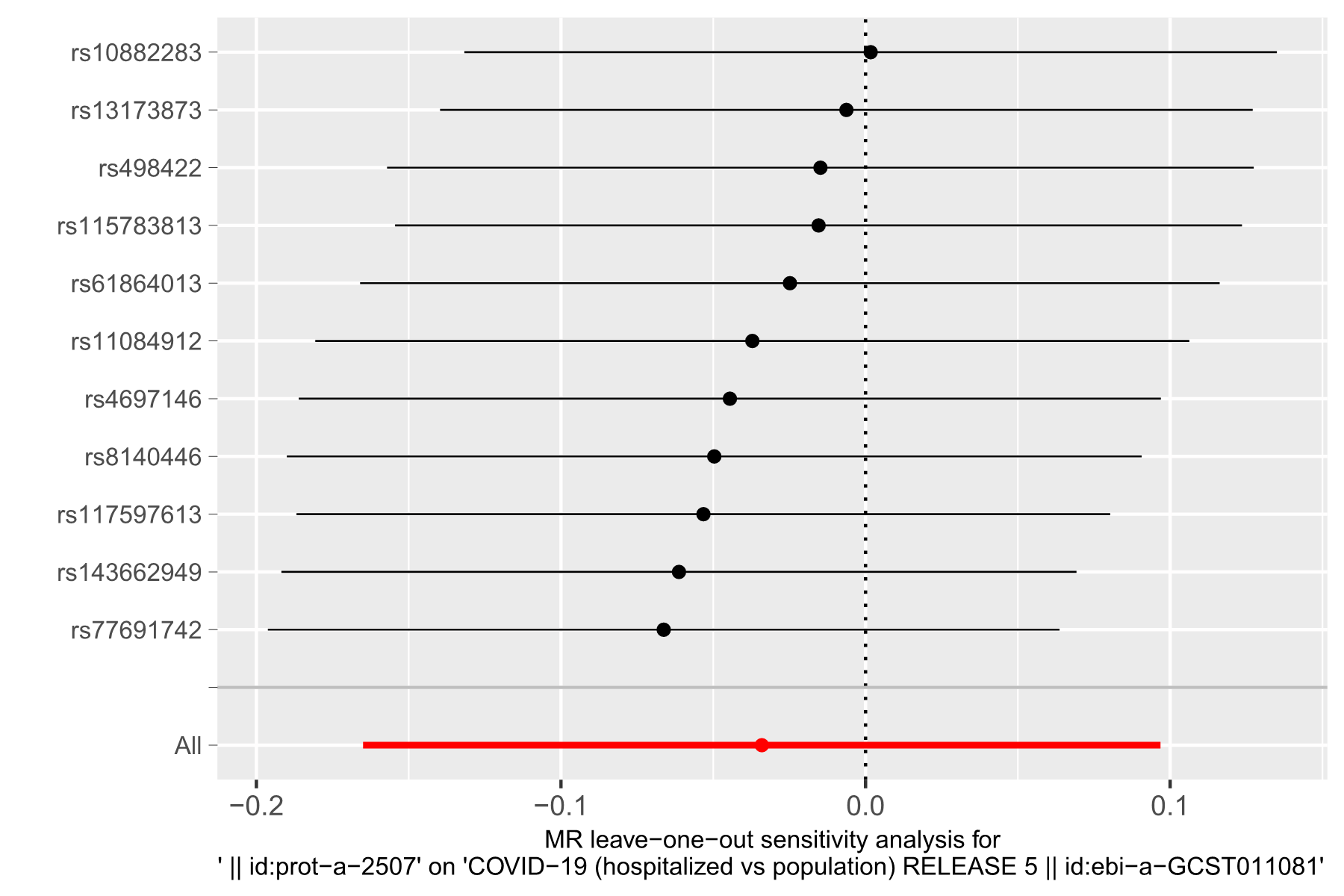


**MR leave-one-out sensitivity analysis for RBP4 || id: prot-a-2507 on COVID-19 hospitalization || id: ebi-a-GCST011081**

**Figure.S15 MR leave-one-out sensitivity analysis for RBP4 on COVID-19 hospitalization. Circles indicate MR estimates for RBP4 on COVID-19 hospitalization using inverse-variance weighted fixed-effect method if each single nucleotide polymorphism was omitted. The bars indicate the CI. MR indicates Mendelian randomization.**

**RBP4 on COVID-19 severity**


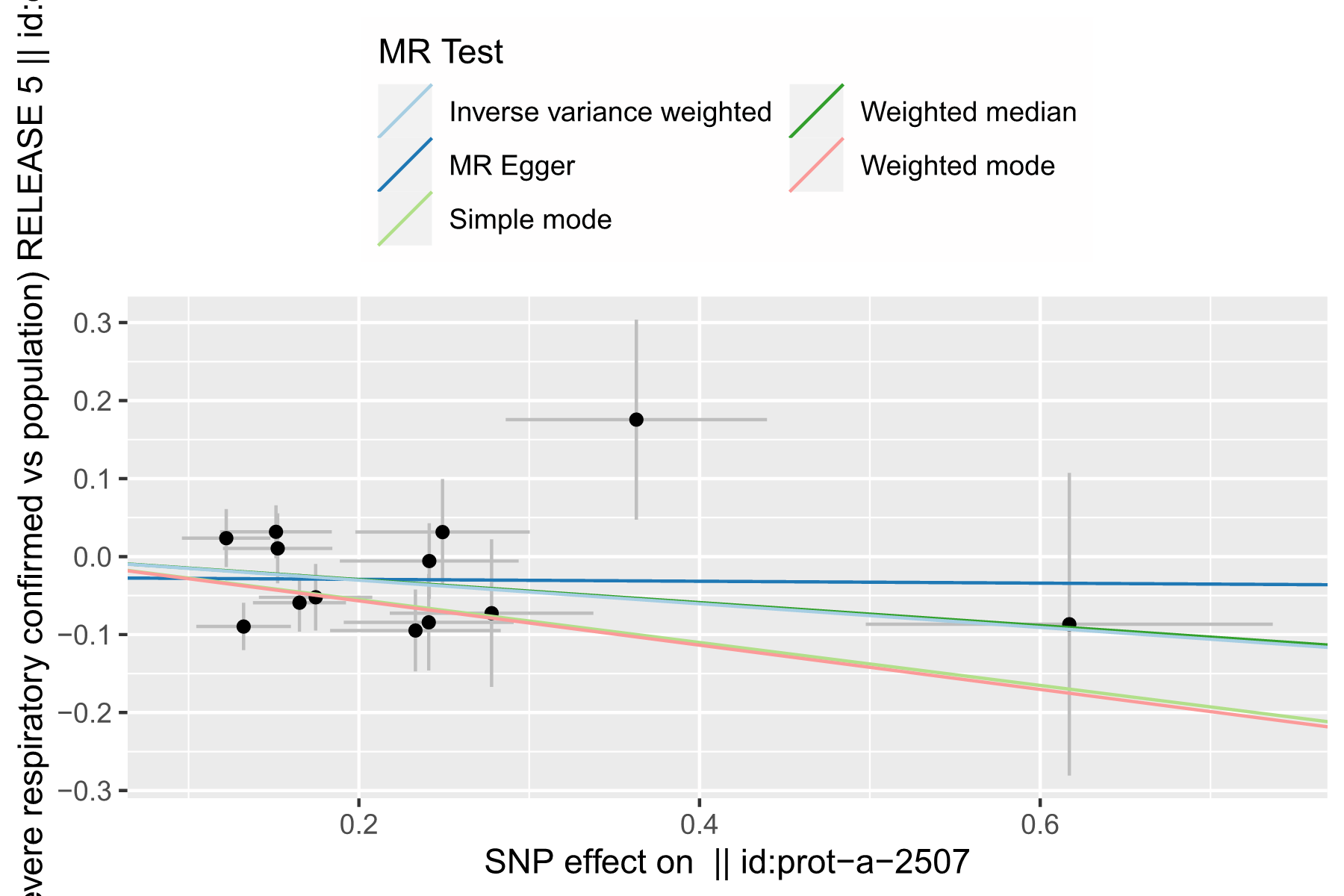


**SNP effect on COVID-19 severity || id: ebi-a-GCST011075**

**SNP effect on RBP4 || id: prot-a-2507**

**Figure.S16** **Scatter plot to visualize the causal effect of RBP4 on COVID-19 severity. The slope of the straight line indicates the magnitude of the causal association. IVW indicates inverse-variance weighted, and MR, Mendelian randomization.**


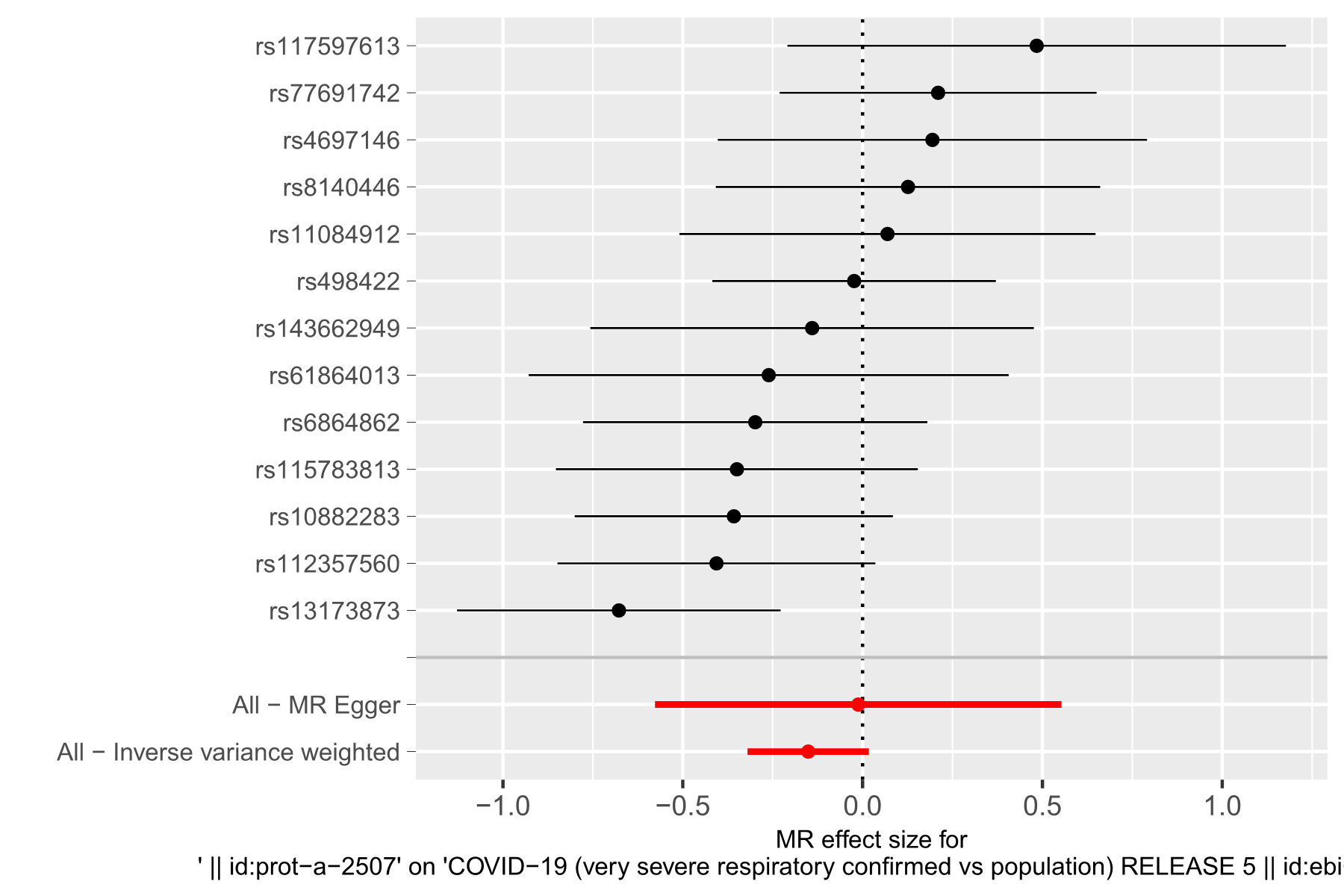


**MR effect size for RBP4 || id: prot-a-2507 on COVID-19 severity || id: ebi-a-GCST011075**

**Figure.S17 Fixed-effect IVW analysis and of the causal association of RBP4 on COVID-19 severity. The black dots and bars indicated the causal estimate and 95% CI using each SNP. The red dot and bar indicated the overall estimate and 95% CI meta-analyzed by fixed-effect inverse variance weighted method and MR-Egger method.**


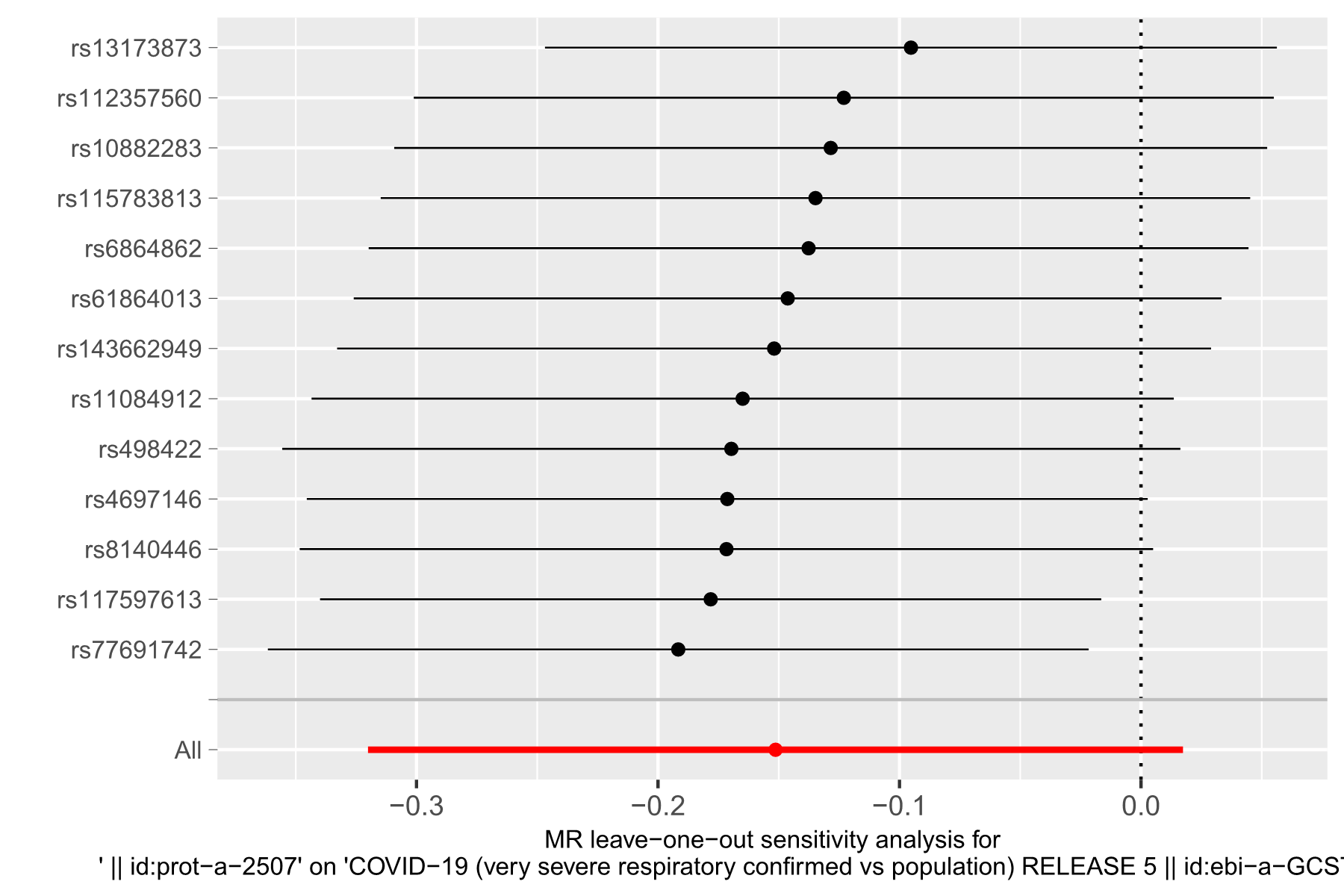


**MR leave-one-out sensitivity analysis for RBP4 || id: prot-a-2507 on COVID-19 severity || id: ebi-a-GCST011075**

**Figure.S18 MR leave-one-out sensitivity analysis for** **RBP4 on COVID-19 severity. Circles indicate MR estimates for RBP4 on COVID-19 severity using inverse-variance weighted fixed-effect method if each single nucleotide polymorphism was omitted. The bars indicate the CI. MR indicates Mendelian randomization.**

**RDH16 on COVID-19 susceptibility**

**SNP effect on COVID-19 susceptibility || id: ebi-a-GCST011072**


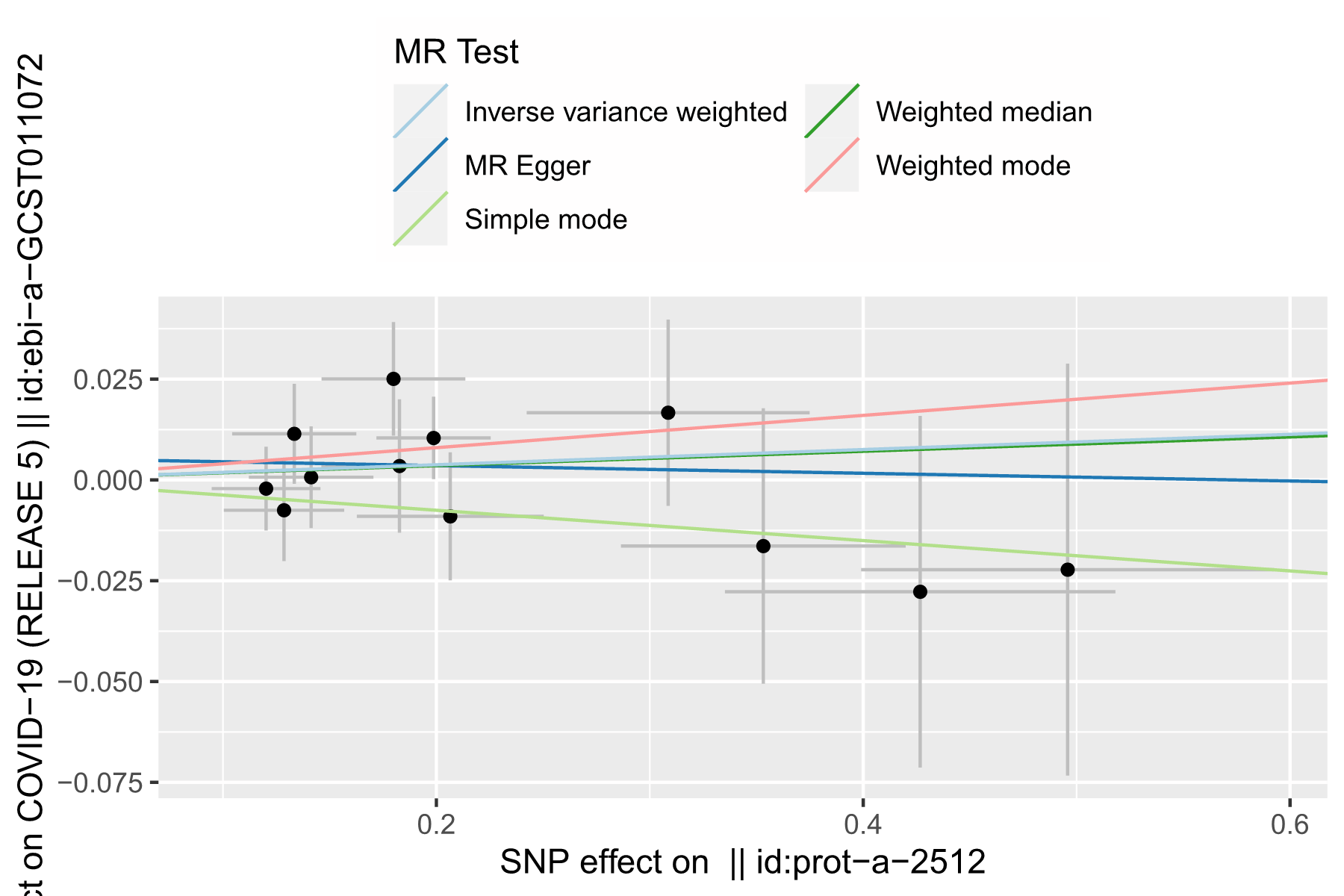


**SNP effect on RDH16 || id: prot-a-2512**

**Figure.S19 Scatter plot to visualize the causal effect of RDH16 on COVID-19 susceptibility. The slope of the straight line indicates the magnitude of the causal association. IVW indicates inverse-variance weighted, and MR, Mendelian randomization.**


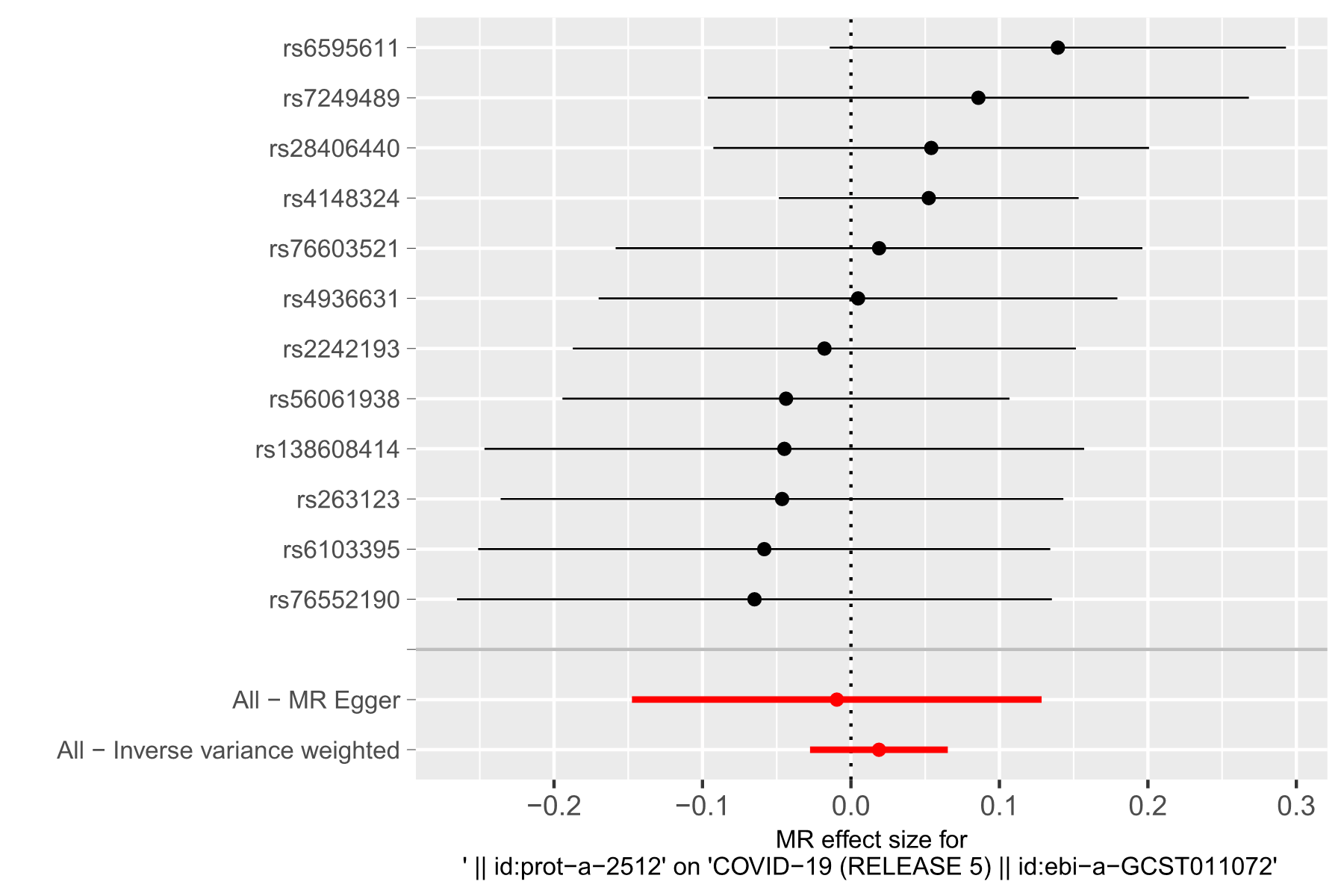


**MR effect size for RDH16 || id: prot-a-2512 on COVID-19 susceptibility || id: ebi-a-GCST011072**

**Figure.S20 Fixed-effect IVW analysis and of the causal association of RDH16 on COVID-19 susceptibility. The black dots and bars indicated the causal estimate and 95% CI using each SNP. The red dot and bar indicated the overall estimate and 95% CI meta-analyzed by fixed-effect inverse variance weighted method and MR-Egger method.**


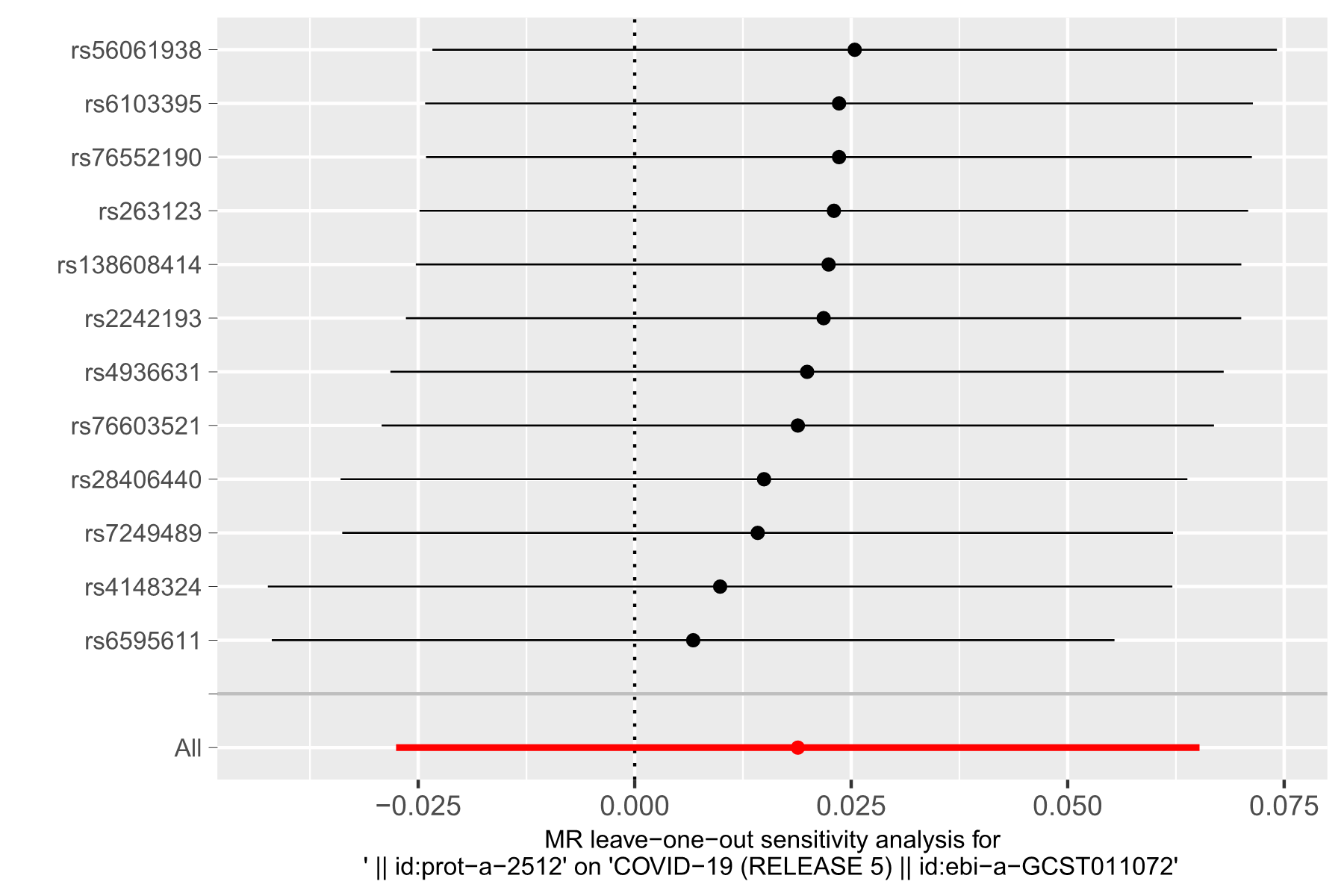


**MR leave-one-out sensitivity analysis for RDH16 || id: prot-a-2512 on COVID-19 susceptibility || id: ebi-a-GCST011072**

**Figure.S21 MR leave-one-out sensitivity analysis for RDH16 on COVID-19 susceptibility. Circles indicate MR estimates for RDH16 on COVID-19 susceptibility using inverse-variance weighted fixed-effect method if each single nucleotide polymorphism was omitted. The bars indicate the CI. MR indicates Mendelian randomization.**

**RDH16 on COVID-19 hospitalization**


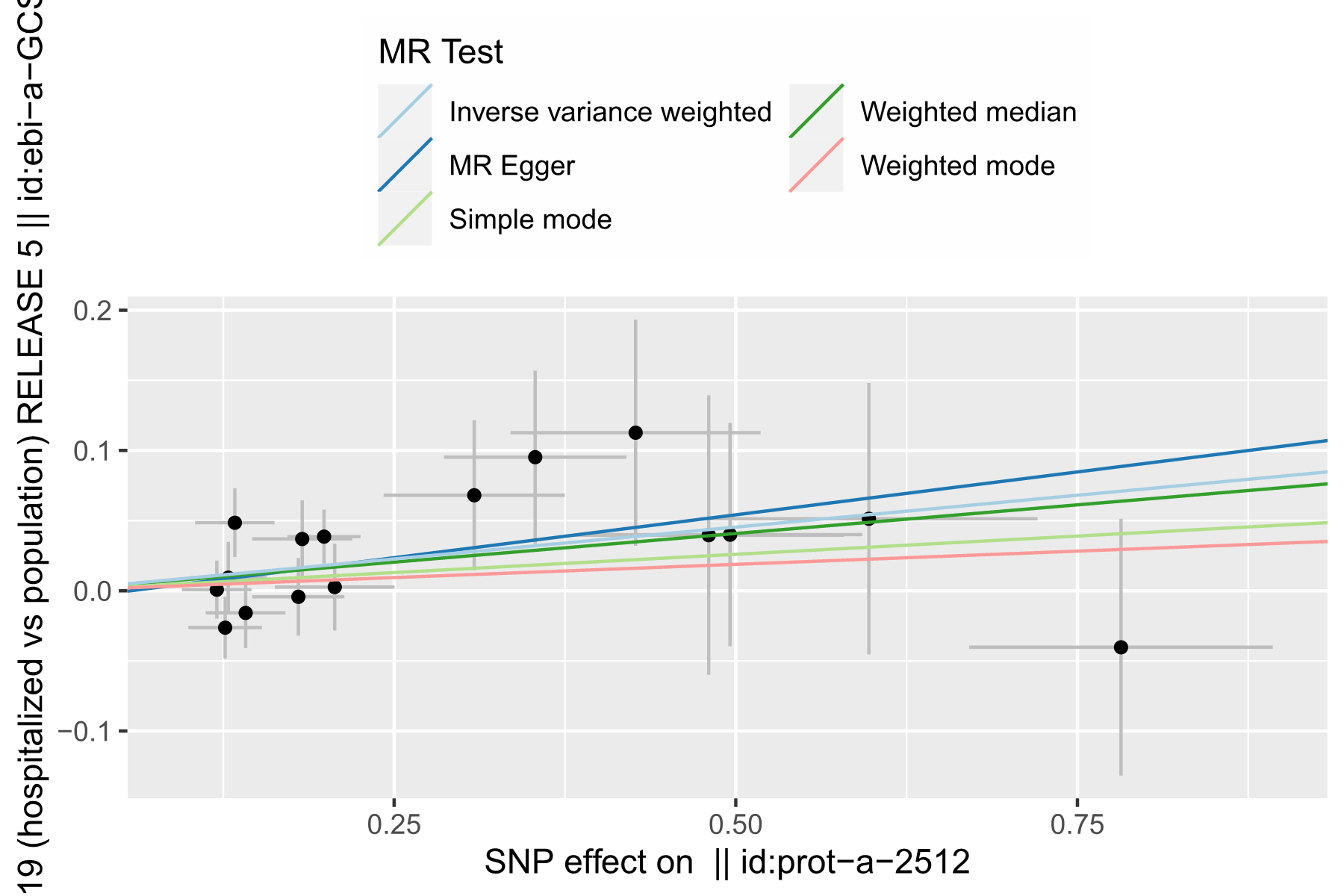


**SNP effect on RDH16 || id: prot-a-2512**

**SNP effect on COVID-19 hospitalization || id: ebi-a-GCST011081**

**Figure.S22 Scatter plot to visualize the causal effect of RDH16 on COVID-19 hospitalization. The slope of the straight line indicates the magnitude of the causal association. IVW indicates inverse-variance weighted, and MR, Mendelian randomization.**


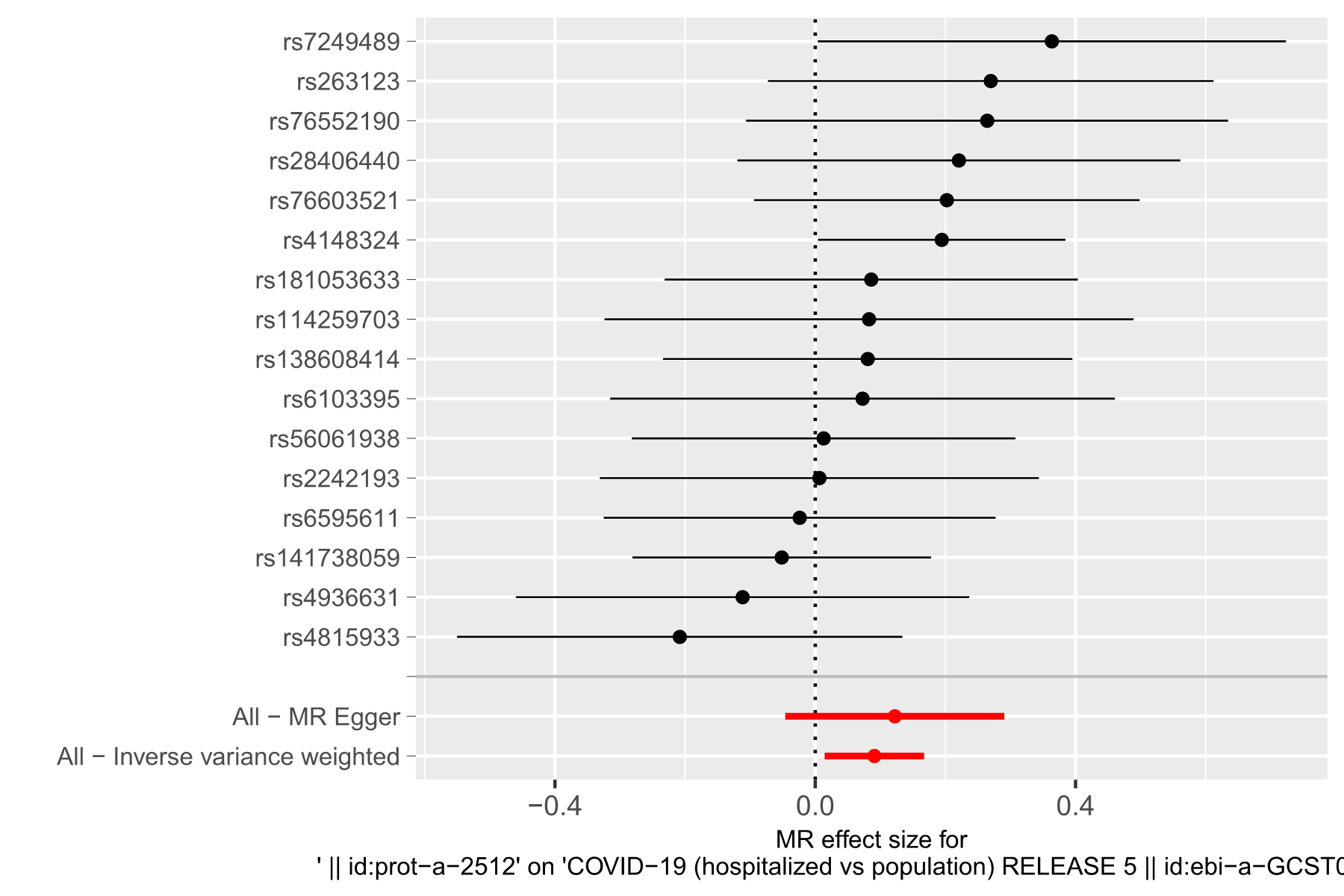


**MR effect size for RDH16 || id: prot-a-2512 on COVID-19 hospitalization || id: ebi-a-GCST011081**

**Figure.S23 Fixed-effect IVW analysis and of the causal association of RDH16 on COVID-19 hospitalization. The black dots and bars indicated the causal estimate and 95% CI using each SNP. The red dot and bar indicated the overall estimate and 95% CI meta-analyzed by fixed-effect inverse variance weighted method and MR-Egger method.**


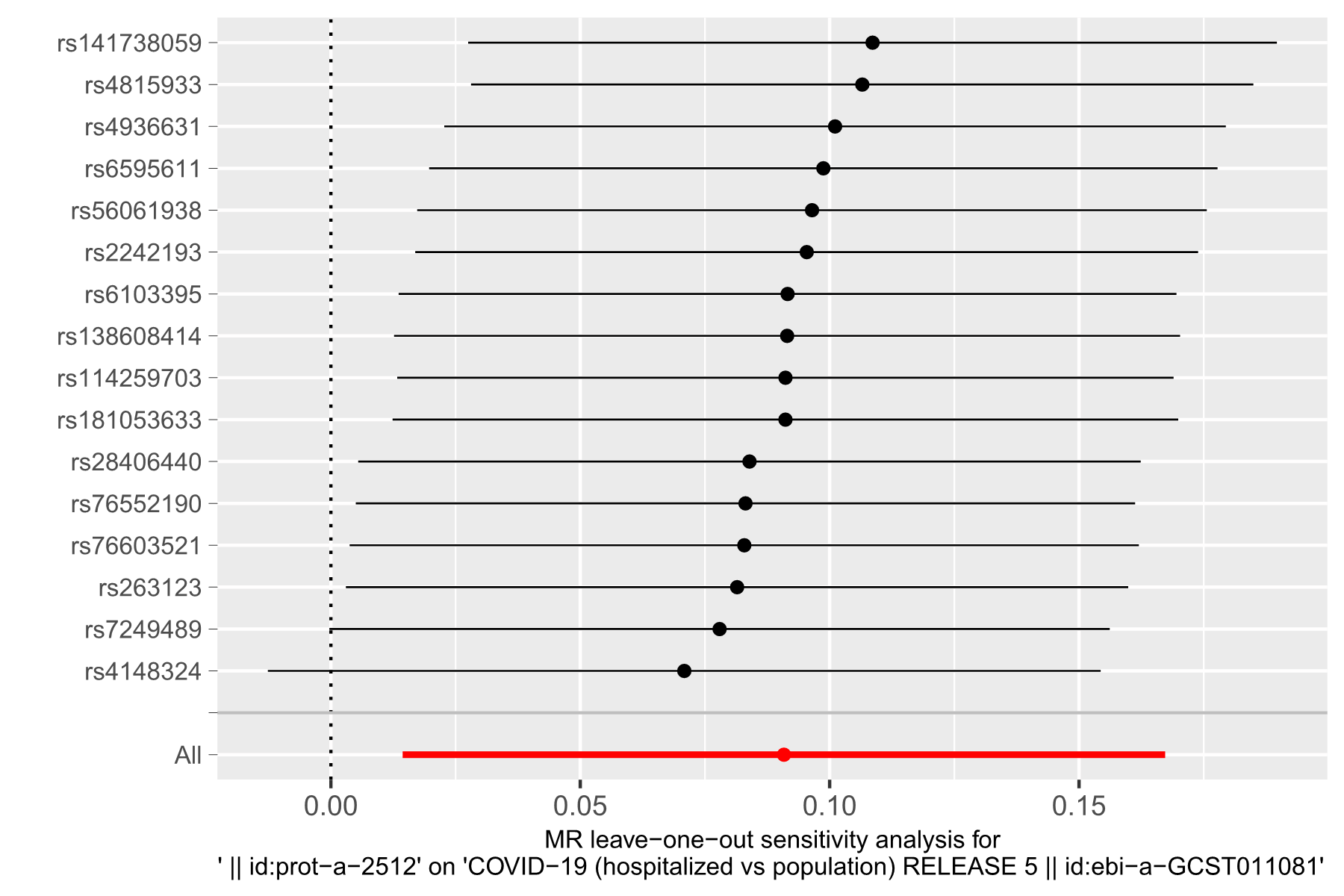


**MR leave-one-out sensitivity analysis for RDH16 || id: prot-a-2512 on COVID-19 hospitalization || id: ebi-a-GCST011081**

**Figure.S24 MR leave-one-out sensitivity analysis for** **RDH16 on COVID-19 hospitalization. Circles indicate MR estimates for RDH16 on COVID-19 hospitalization using inverse-variance weighted fixed-effect method if each single nucleotide polymorphism was omitted. The bars indicate the CI. MR indicates Mendelian randomization.**

**RDH16 on COVID-19 severity**


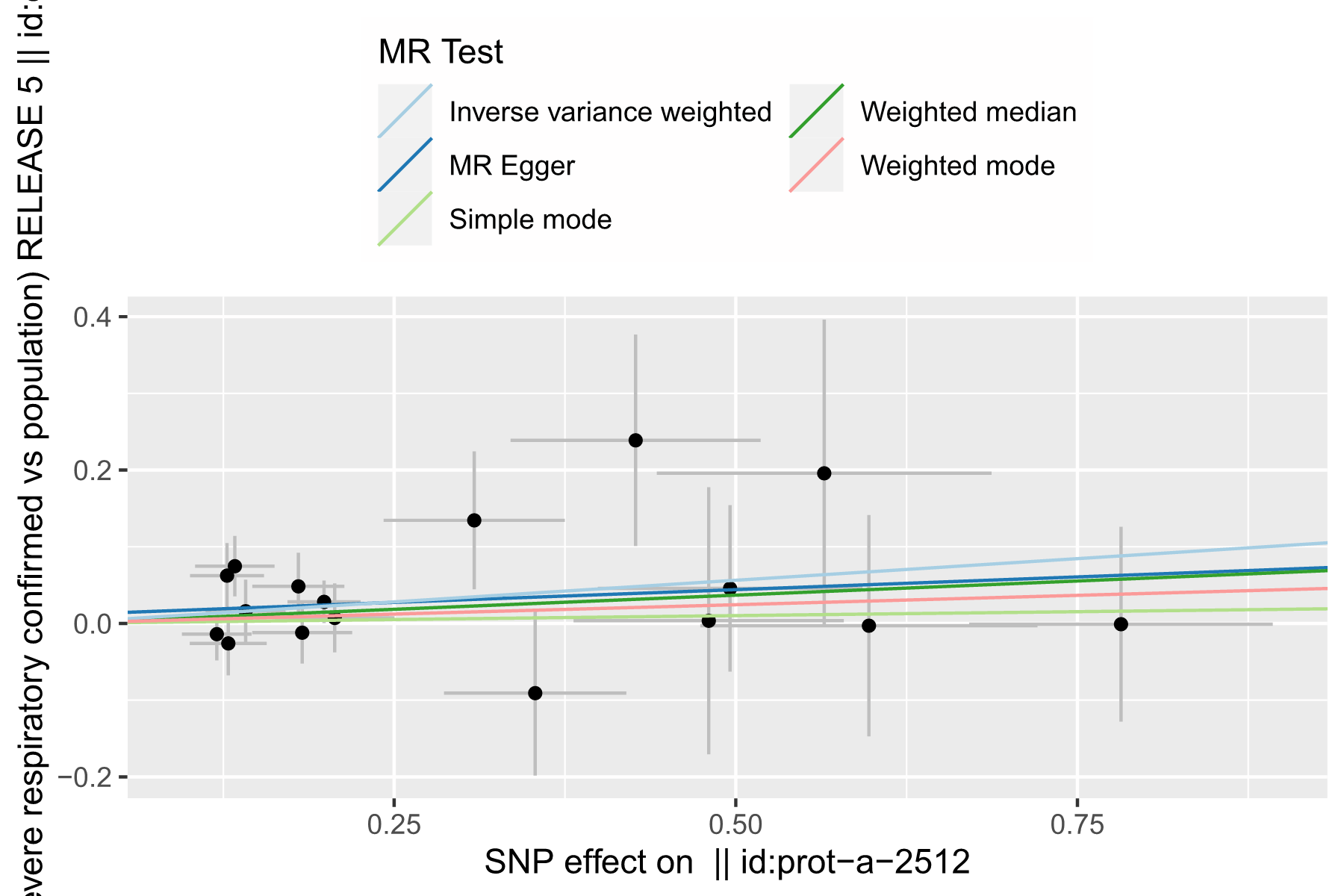


**SNP effect on RDH16 || id: prot-a-2512**

**SNP effect on COVID-19 severity || id: ebi-a-GCST011075**

**Figure.S25 Scatter plot to visualize the causal effect of RDH16 on COVID-19 severity. The slope of the straight line indicates the magnitude of the causal association. IVW indicates inverse-variance weighted, and MR, Mendelian randomization.**


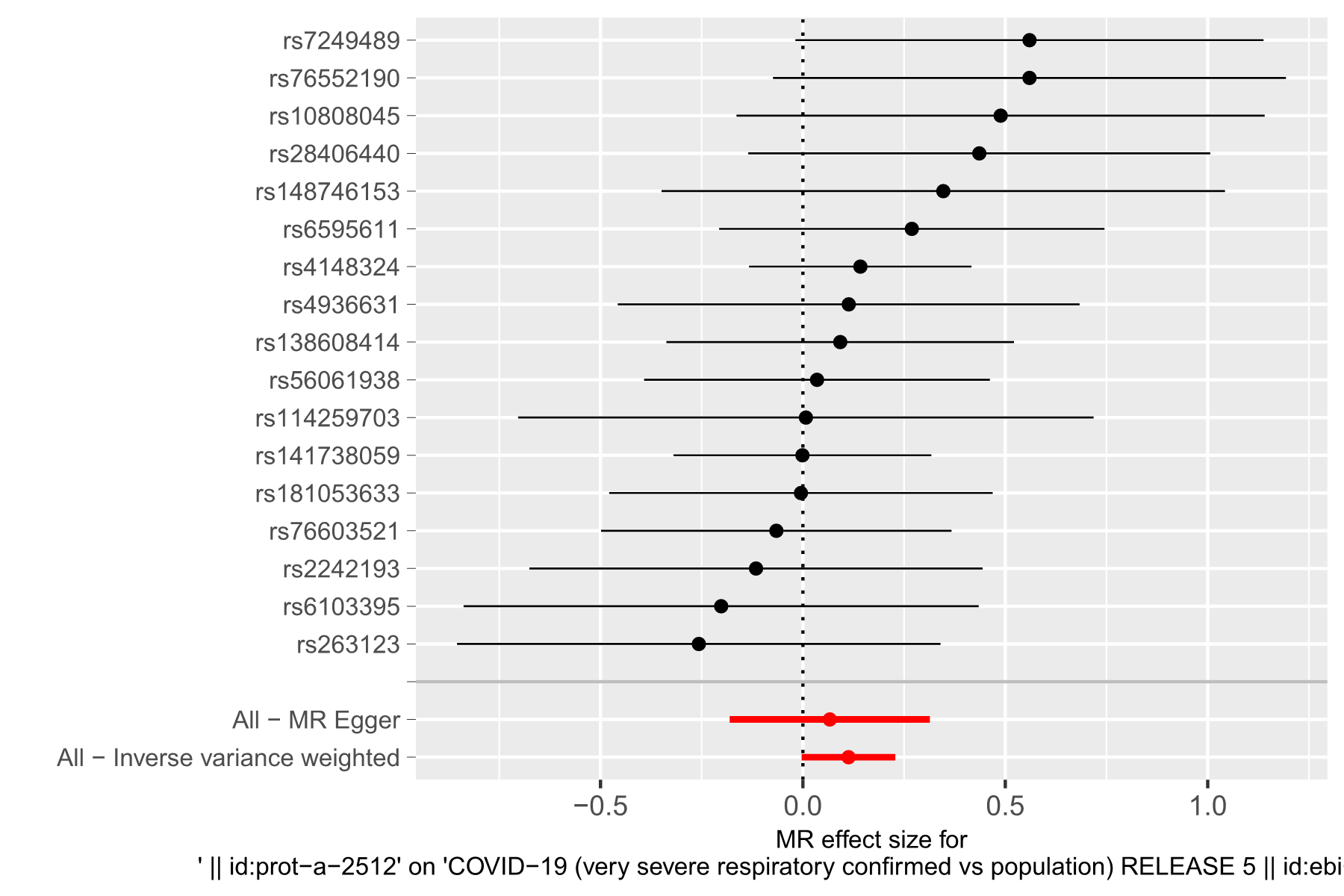


**MR effect size for RDH16 || id: prot-a-2512 on COVID-19 severity || id: ebi-a-GCST011075**

**Figure.S26 Fixed-effect IVW analysis and of the causal association of RDH16 on COVID-19 severity. The black dots and bars indicated the causal estimate and 95% CI using each SNP. The red dot and bar indicated the overall estimate and 95% CI meta-analyzed by fixed-effect inverse variance weighted method and MR-Egger method.**


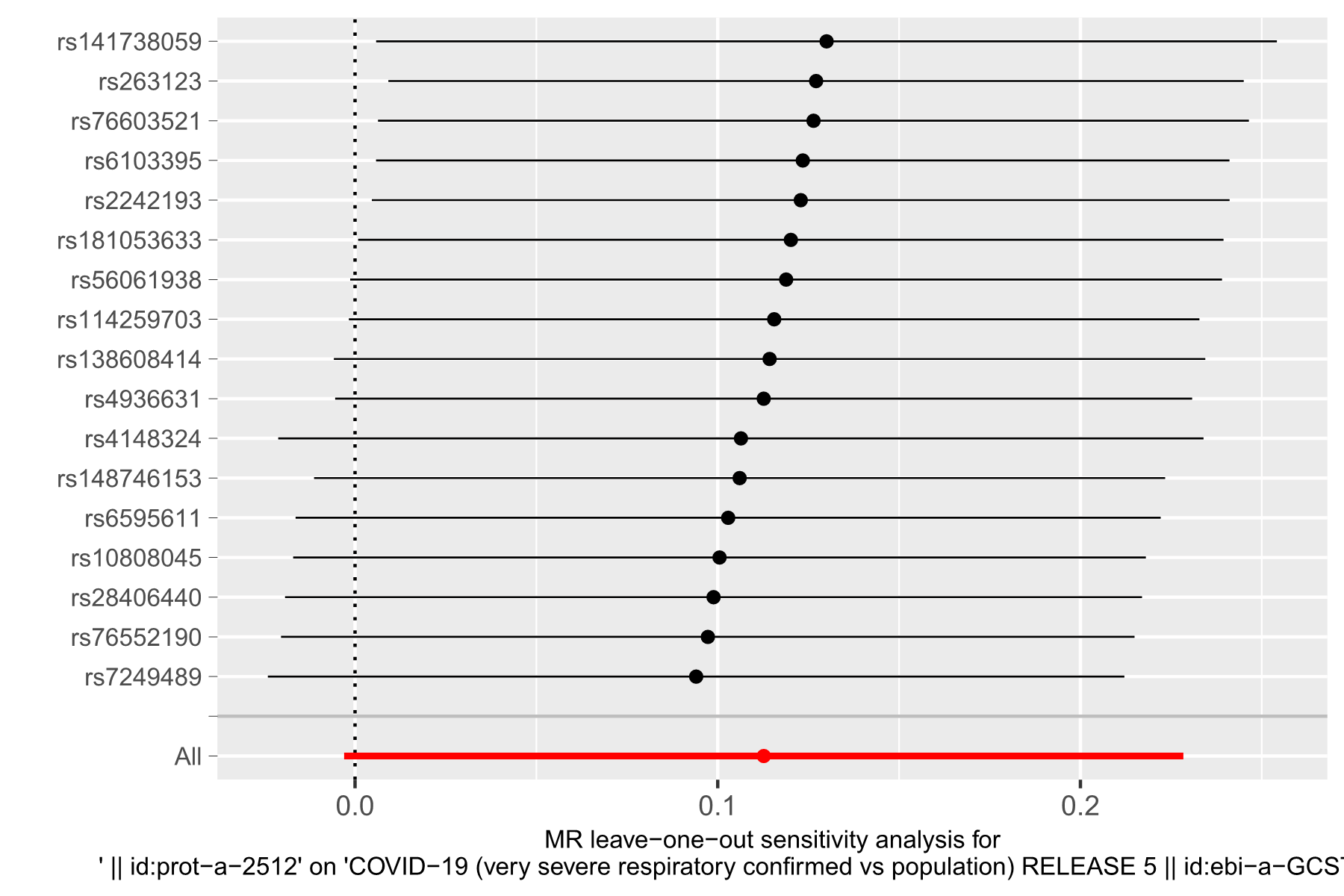


**MR leave-one-out sensitivity analysis for RDH16 || id: prot-a-2512 on COVID-19 severity || id: ebi-a-GCST011075**

**Figure.S27 MR leave-one-out sensitivity analysis for** **RDH16 on COVID-19 severity. Circles indicate MR estimates for RDH16 on COVID-19 severity using inverse-variance weighted fixed-effect method if each single nucleotide polymorphism was omitted. The bars indicate the CI. MR indicates Mendelian randomization.**

**CRABP1 on COVID-19 susceptibility**

**SNP effect on COVID-19 susceptibility || id: ebi-a-GCST011072**


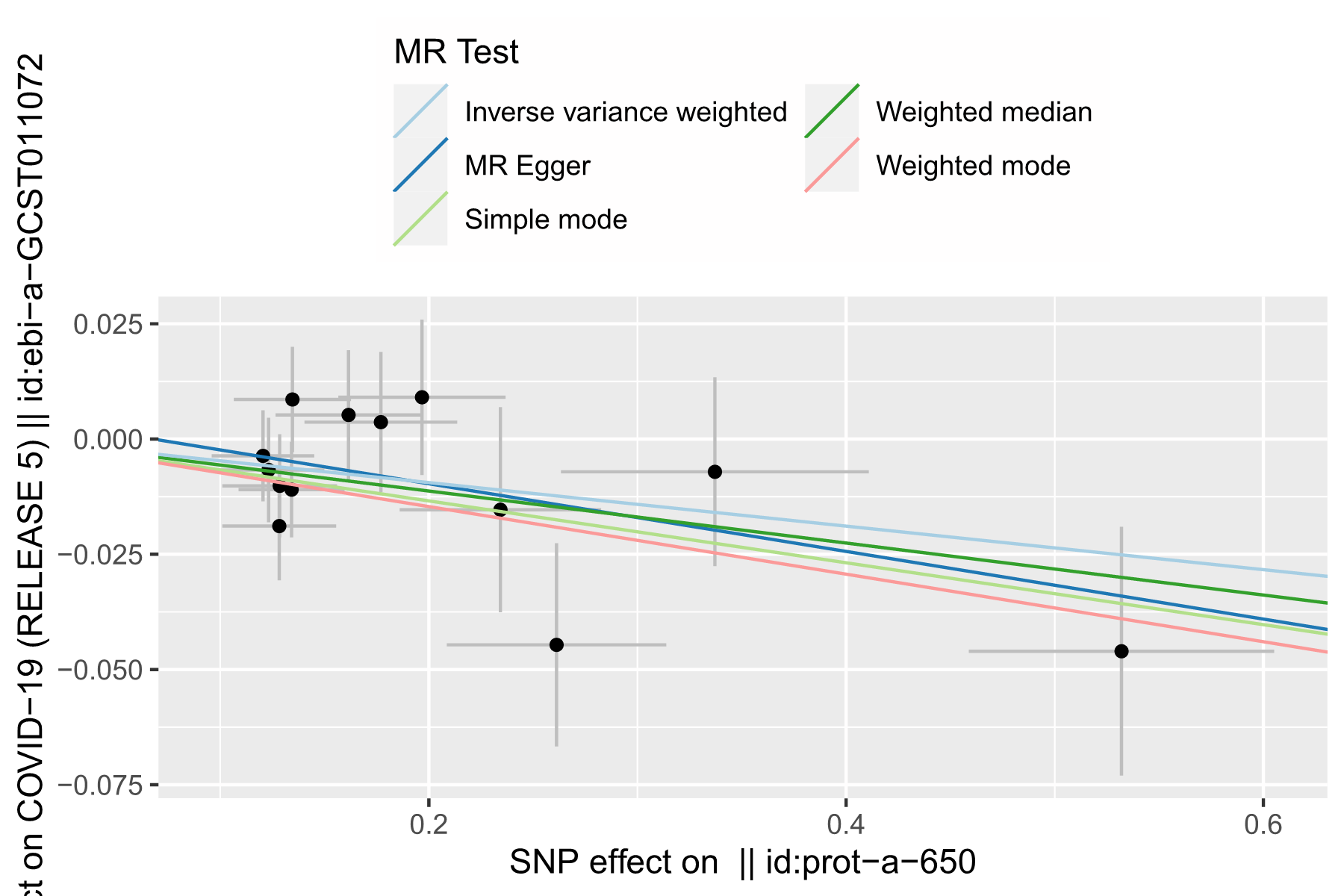


**SNP effect on CRABP1 || id: prot-a-650**

**Figure.S28 Scatter plot to visualize the causal effect of CRABP1 on COVID-19 susceptibility. The slope of the straight line indicates the magnitude of the causal association. IVW indicates inverse-variance weighted, and MR, Mendelian randomization.**


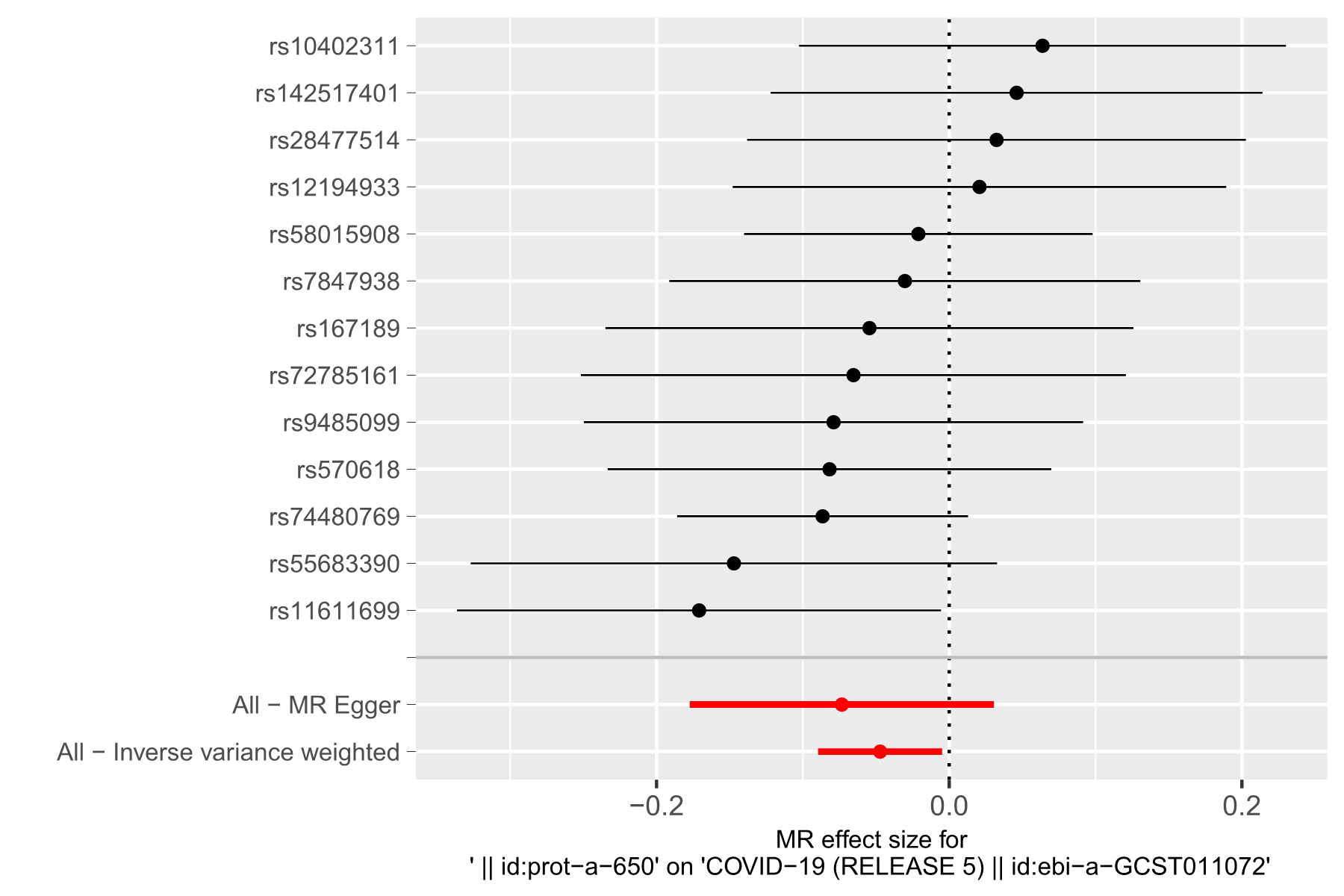


**MR effect size for CRABP1 || id: prot-a-650 on COVID-19 susceptibility || id: ebi-a-GCST011072**

**Figure.S29 Fixed-effect IVW analysis and of the causal association of CRABP1 on COVID-19 susceptibility. The black dots and bars indicated the causal estimate and 95% CI using each SNP. The red dot and bar indicated the overall estimate and 95% CI meta-analyzed by fixed-effect inverse variance weighted method and MR-Egger method.**


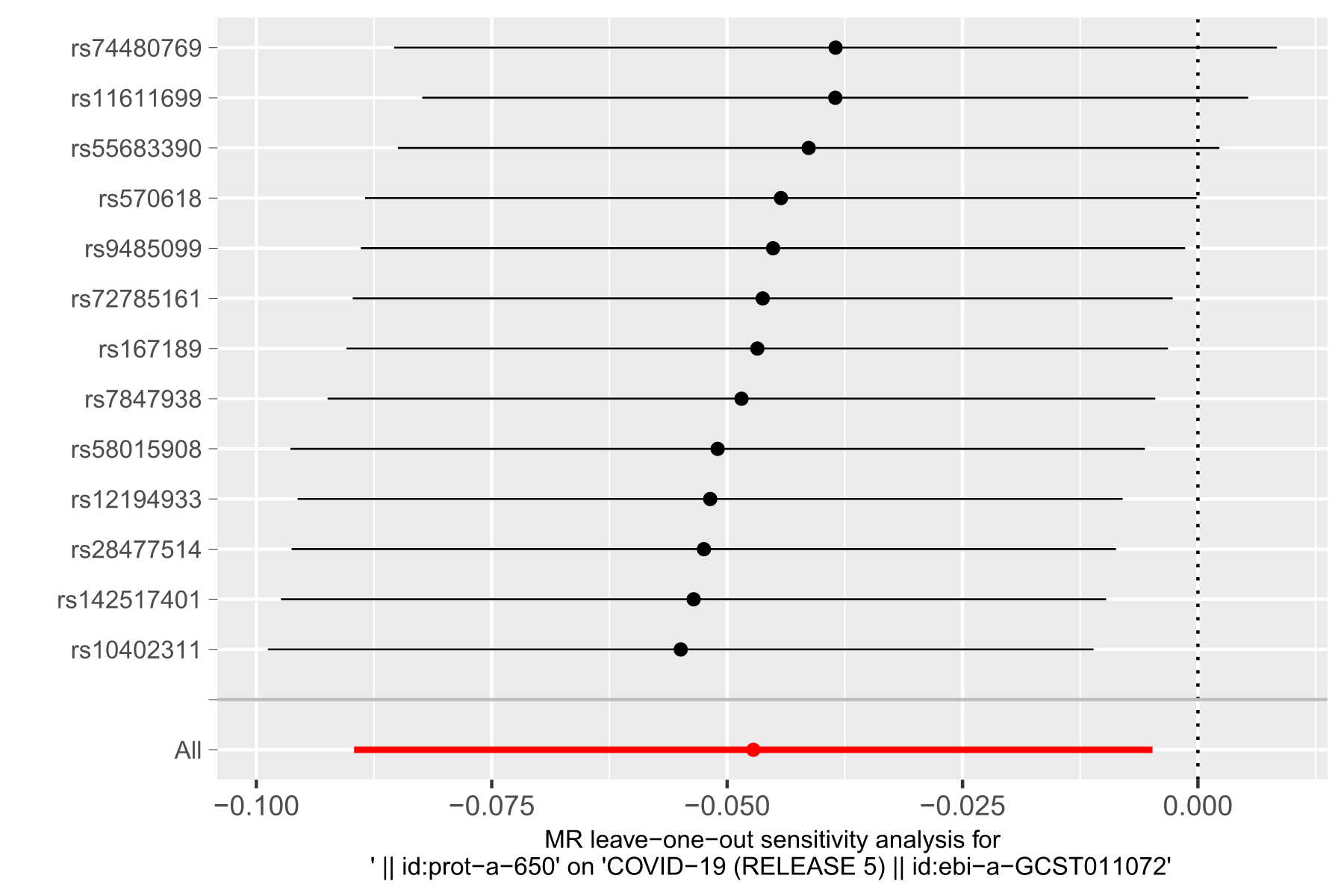


**MR leave-one-out sensitivity analysis for CRABP1 || id: prot-a-650 on COVID-19 susceptibility || id: ebi-a-GCST011072**

**Figure.S30 MR leave-one-out sensitivity analysis for CRABP1 on COVID-19 susceptibility. Circles indicate MR estimates for CRABP1 on COVID-19 susceptibility using inverse-variance weighted fixed-effect method if each single nucleotide polymorphism was omitted. The bars indicate the CI. MR indicates Mendelian randomization.**

**CRABP1 on COVID-19 hospitalization**

**SNP effect on COVID-19 hospitalization || id: ebi-a-GCST011081**


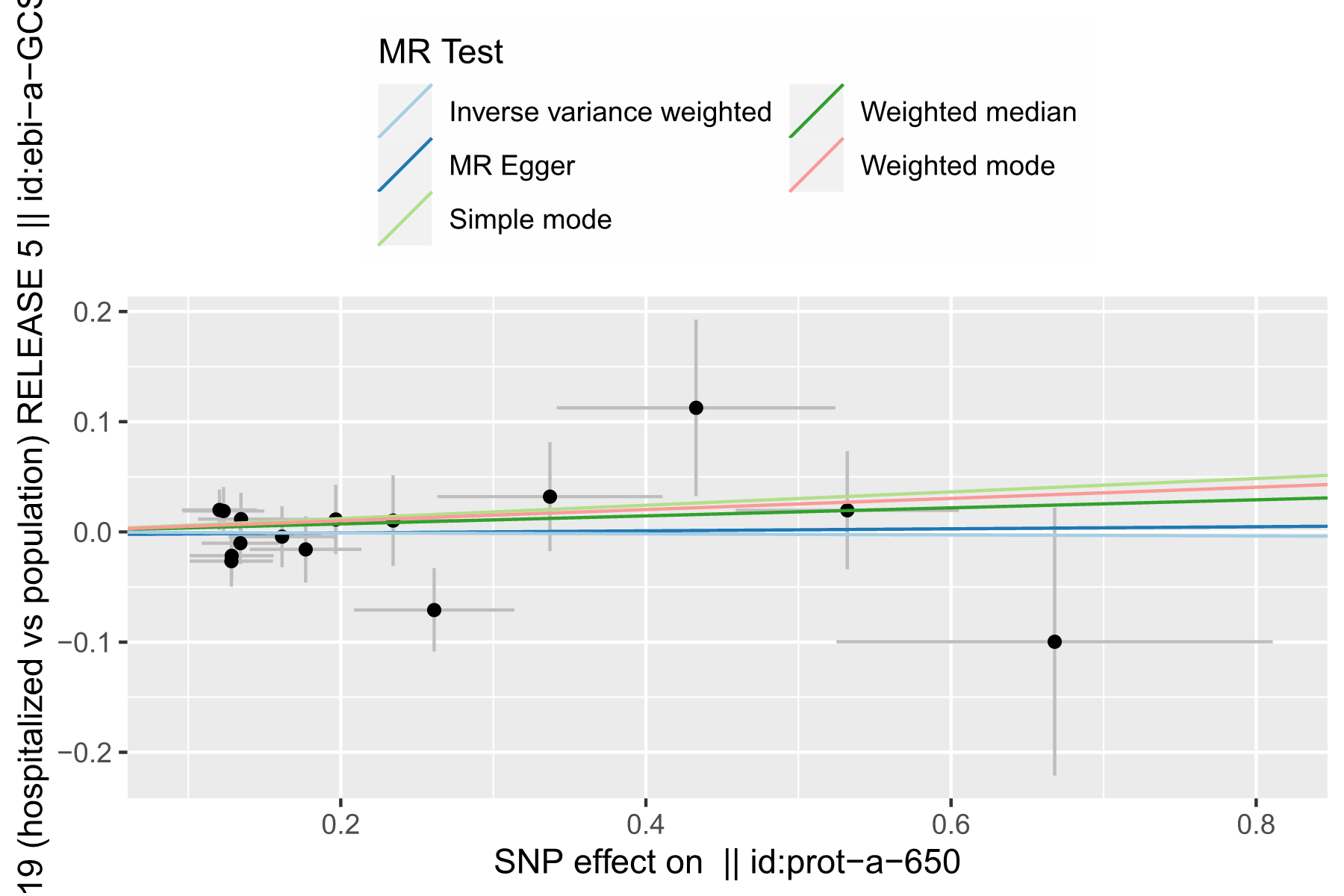


**SNP effect on CRABP1 || id: prot-a-650**

**Figure.S31 Scatter plot to visualize the causal effect of CRABP1 on COVID-19 hospitalization. The slope of the straight line indicates the magnitude of the causal association. IVW indicates inverse-variance weighted, and MR, Mendelian randomization.**


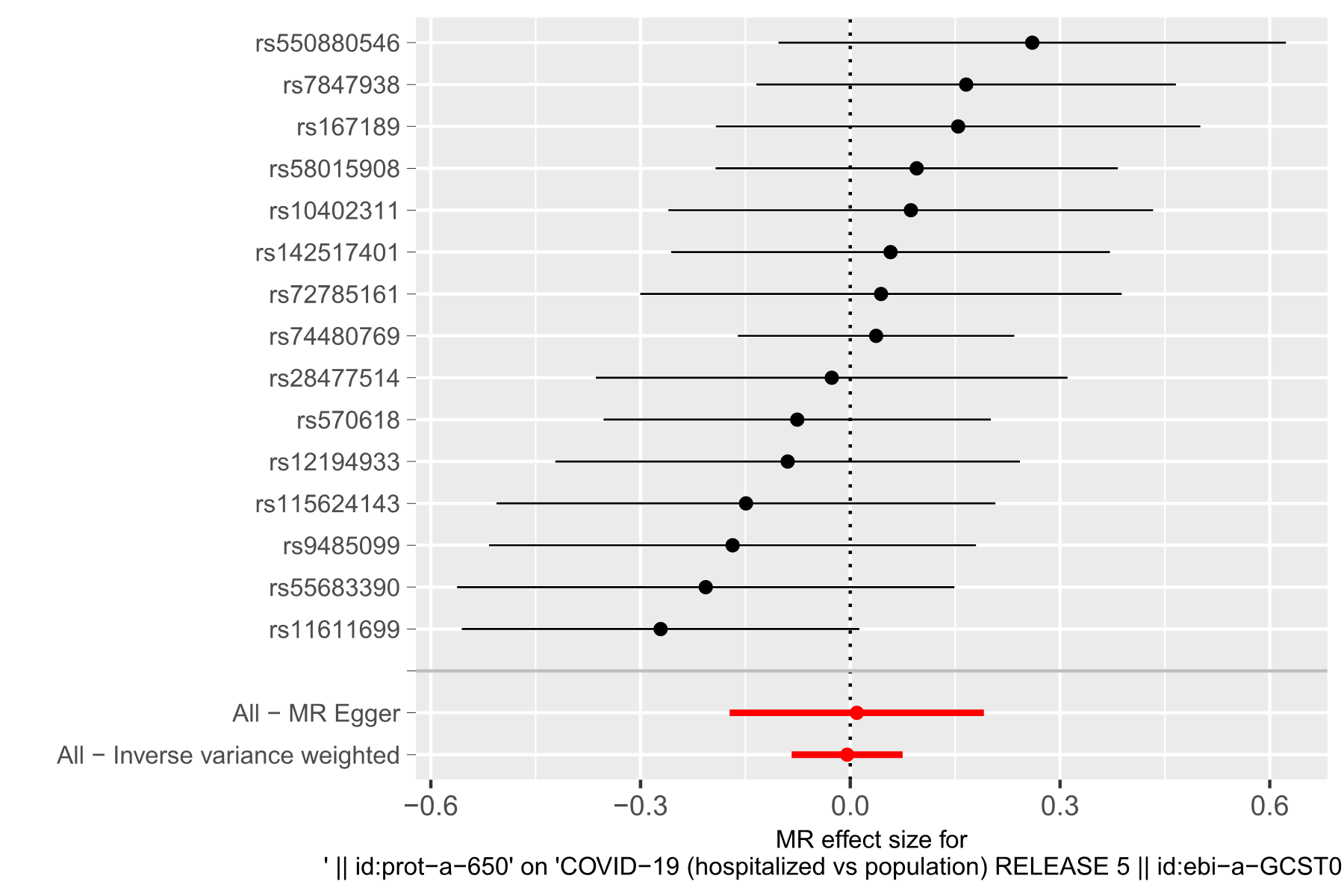


**MR effect size for CRABP1 || id: prot-a-650 on COVID-19 hospitalization || id: ebi-a-GCST011081**

**Figure.S32 Fixed-effect IVW analysis and of the causal association of CRABP1 on COVID-19 hospitalization. The black dots and bars indicated the causal estimate and 95% CI using each SNP. The red dot and bar indicated the overall estimate and 95% CI meta-analyzed by fixed-effect inverse variance weighted method and MR-Egger method.**


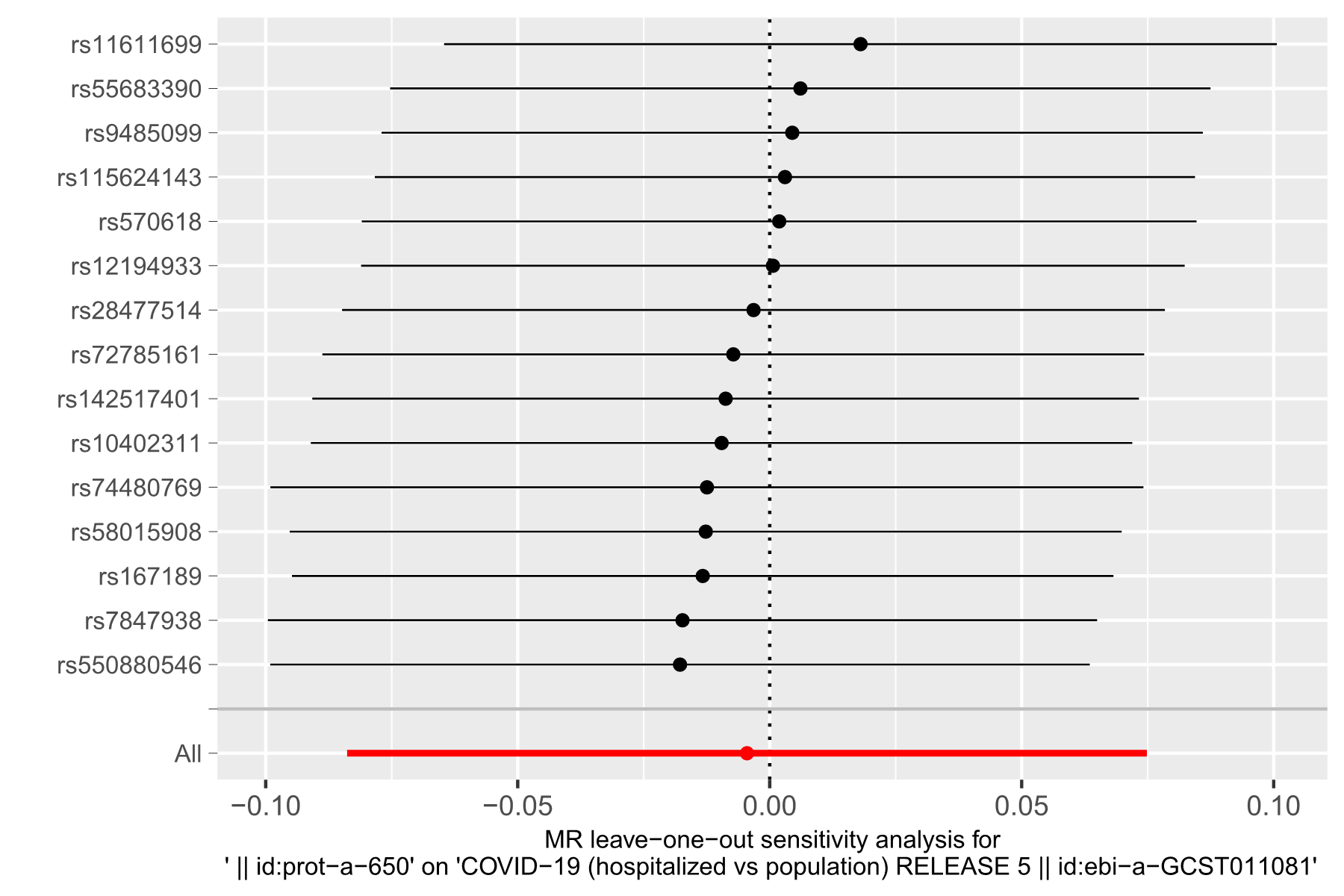


**MR leave-one-out sensitivity analysis for CRABP1 || id: prot-a-650 on COVID-19 hospitalization || id: ebi-a-GCST011081**

**Figure.S33 MR leave-one-out sensitivity analysis for** **CRABP1 on COVID-19 hospitalization. Circles indicate MR estimates for CRABP1 on COVID-19 hospitalization using inverse-variance weighted fixed-effect method if each single nucleotide polymorphism was omitted. The bars indicate the CI. MR indicates Mendelian randomization.**

**CRABP1 on COVID-19 severity**


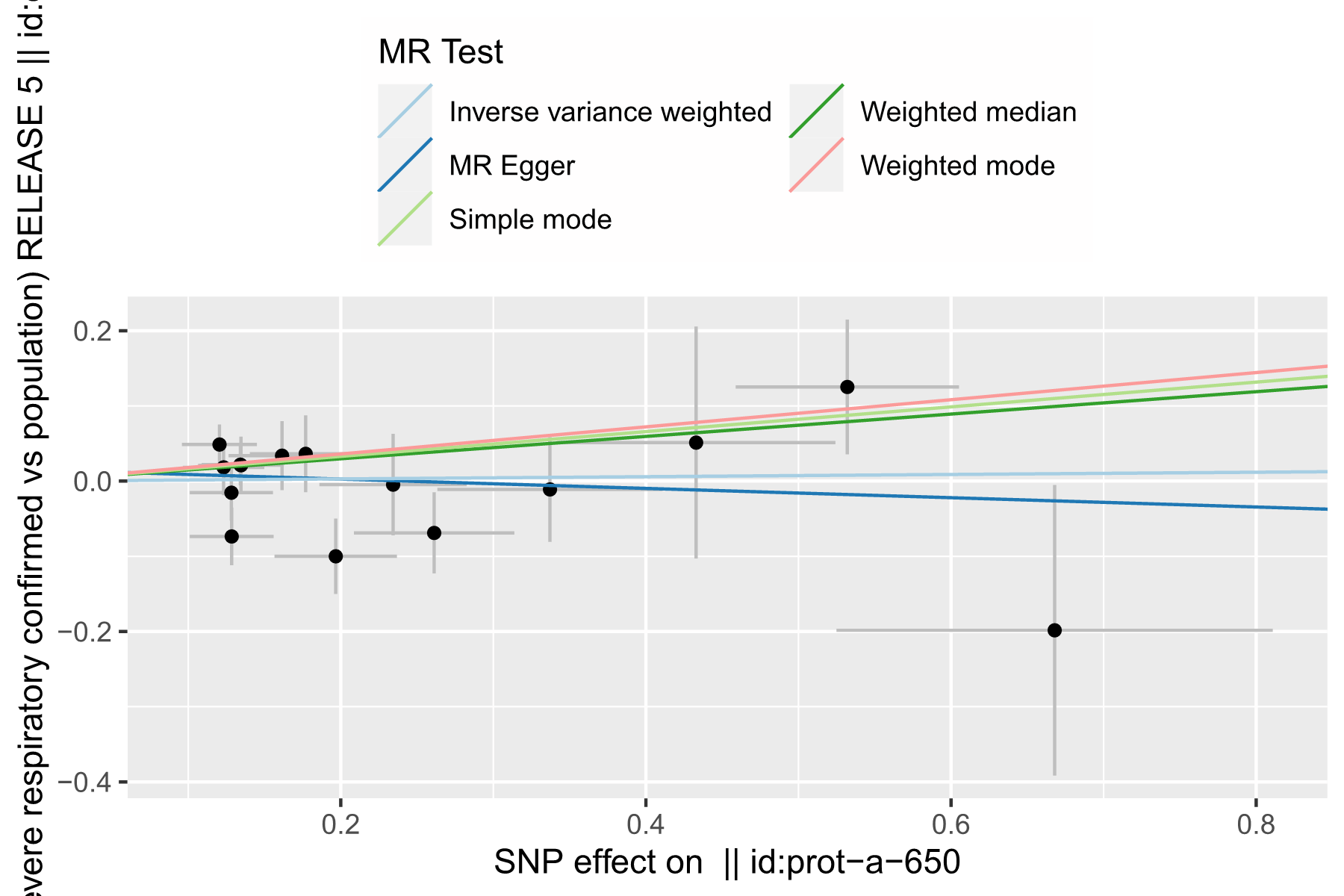


**SNP effect on CRABP1 || id: prot-a-650**

**SNP effect on COVID-19 severity || id: ebi-a-GCST011075**

**Figure.S34 Scatter plot to visualize the causal effect of** **CRABP1 on COVID-19 severity. The slope of the straight line indicates the magnitude of the causal association. IVW indicates inverse-variance weighted, and MR, Mendelian randomization.**


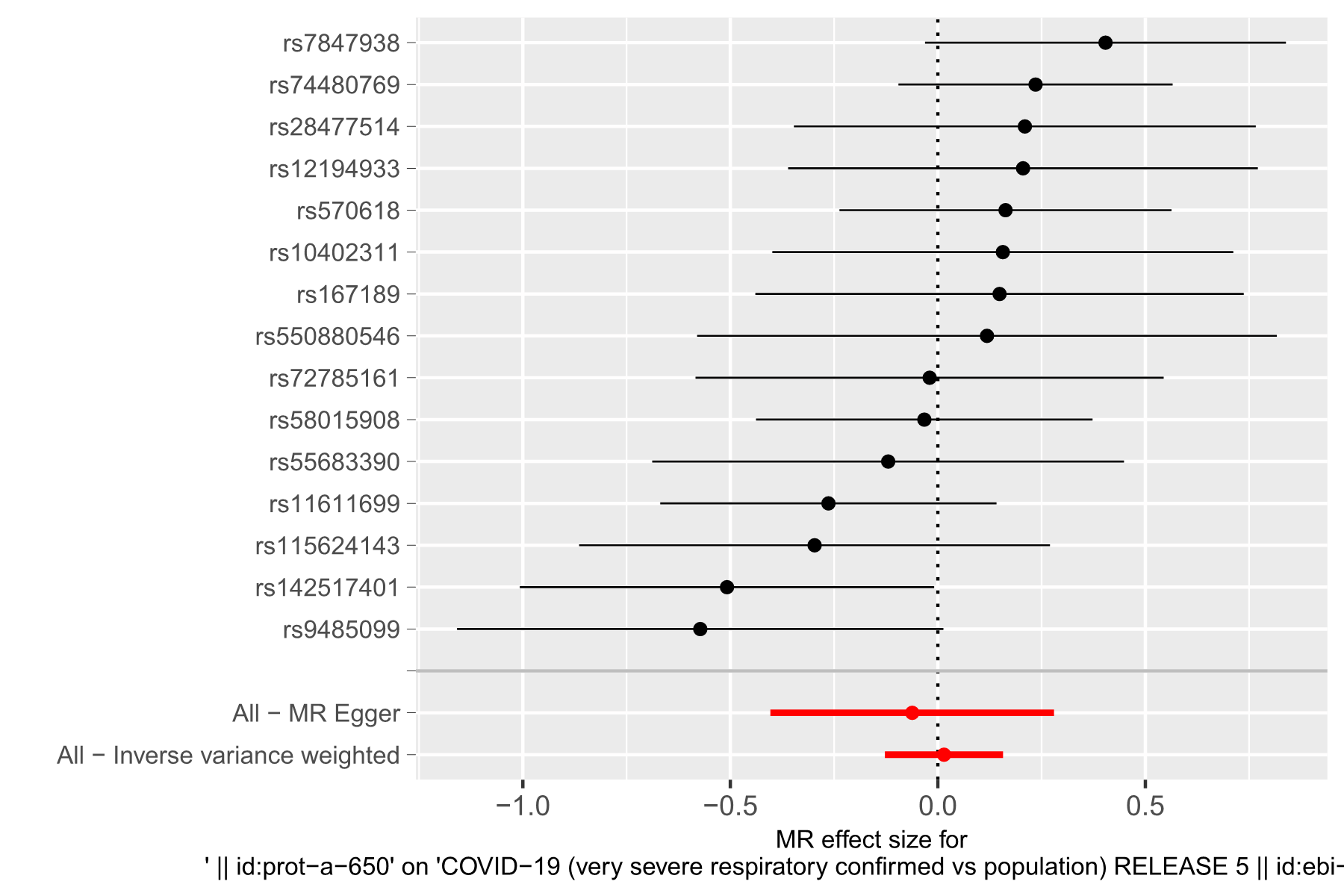


**MR effect size for CRABP1 || id: prot-a-650 on COVID-19 severity || id: ebi-a-GCST011075**

**Figure.S35 Fixed-effect IVW analysis and of the causal association of CRABP1 on COVID-19 severity. The black dots and bars indicated the causal estimate and 95% CI using each SNP. The red dot and bar indicated the overall estimate and 95% CI meta-analyzed by fixed-effect inverse variance weighted method and MR-Egger method.**


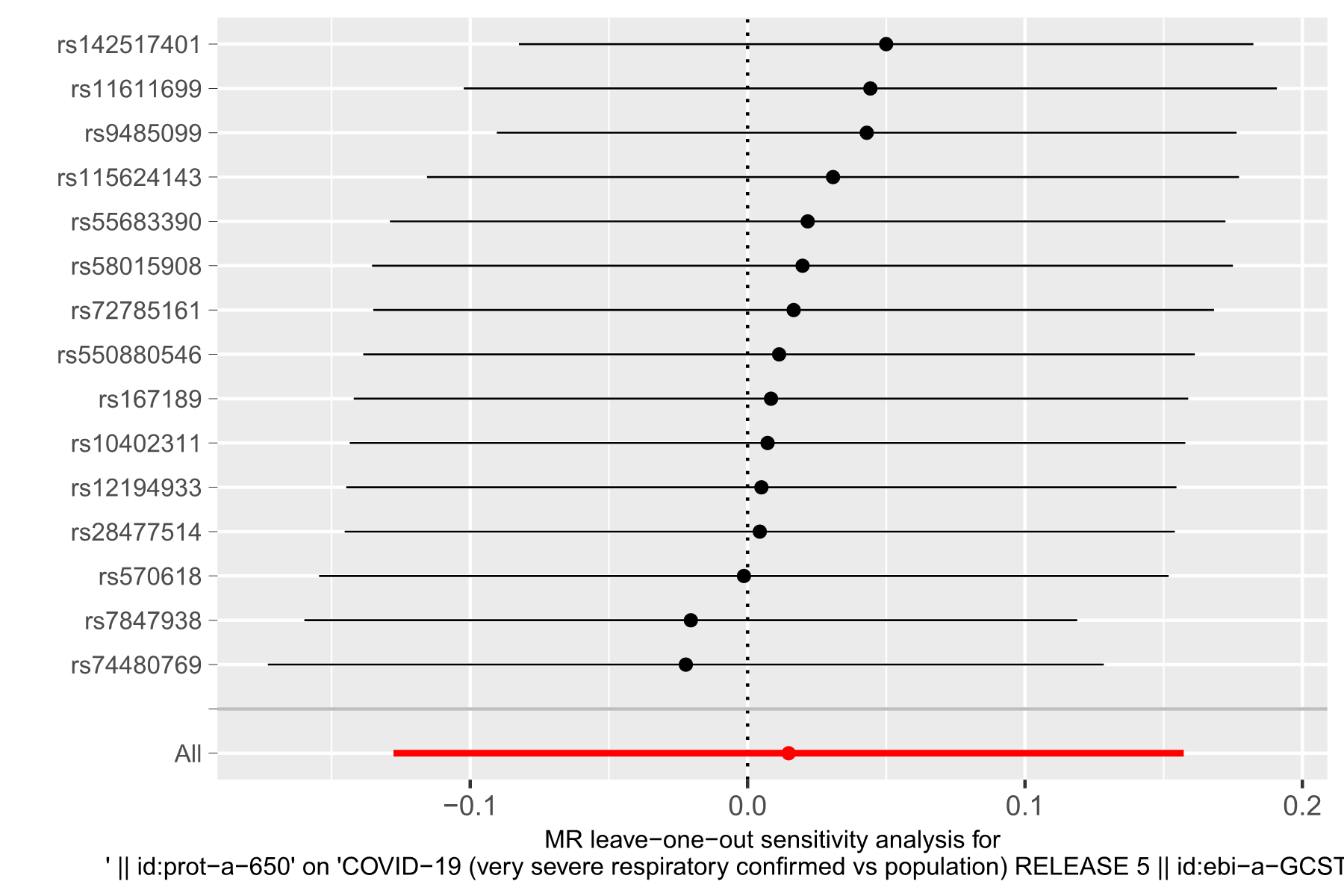


**MR leave-one-out sensitivity analysis for CRABP1 || id: prot-a-650 on COVID-19 severity || id: ebi-a-GCST011075**

**Figure.S36 MR leave-one-out sensitivity analysis for** **CRABP1 on COVID-19 severity. Circles indicate MR estimates for CRABP1 on COVID-19 severity using inverse-variance weighted fixed-effect method if each single nucleotide polymorphism was omitted. The bars indicate the CI. MR indicates Mendelian randomization.**
