# Supplementary material for: Retinol and retinol binding protein 4 levels and COVID-19: a Mendelian randomization study
Source: BMC Pulm Med. 2024 Apr 26;24:206. doi: 10.1186/s12890-024-03013-w (PMC11046857; doi:10.1186/s12890-024-03013-w)
Supplement: Supplementary file 2 — Supplementary Material 2 [file 12890_2024_3013_MOESM2_ESM.docx]

**Supplementary documents for**

**Retinol and Retinol Binding Protein 4 Levels and COVID-19: A Mendelian Randomization Study**

**Supplementary Table 1.** Information on outcome data sources

**Supplementary Table 2.** Information on instrumental variables

**Supplementary Table 1.** Information on outcome data sources

| **Exposure or Outcome** | **Ethnicity** | **Total population** | **Cases/controls** | **Source** |
| --- | --- | --- | --- | --- |
| COVID-19 susceptibility | European | 1,058,410 | 31,562/1,026,848 | PMID: 32404885 |
| COVID-19 hospitalization | European | 1,887,658 | 9,986/1,877,672 | PMID: 32404885 |
| COVID-19 severity | European | 1,388,342 | 5,101/1,383,241 | PMID: 32404885 |
| Retinol | European | 62,991 | NA | UK Biobank |
| Retinol-binding protein 4 | European | 3,301 | NA | PMID: 29875488 |
| Retinol dehydrogenase 16 | European | 3,301 | NA | PMID: 29875488 |
| Cellular retinoic acid-binding protein 1 | European | 3,301 | NA | PMID: 29875488 |

**Supplementary Table 2.** Information on instrumental variables.

| **Exposure** | **SNP** | **Chr** | **Position** | **EA/OA** | **Gene** | **EAF** | **Beta** | **SE** | **P value** | **F-statistic** | **R^2^** |
| --- | --- | --- | --- | --- | --- | --- | --- | --- | --- | --- | --- |
| Retinol | rs117219913 | 16 | 61692702 | C/T | CDH8 | 0.0783 | 0.0477 | 0.0104 | 4.40 x 10^-6^ | 21.10 | 3.35 x 10^-4^ |
|  | rs117669768 | 14 | 100960862 | A/G | WDR25 | 0.0382 | 0.0790 | 0.0148 | 9.00 x 10^-8^ | 28.58 | 4.54 x 10^-4^ |
|  | rs12119164 | 1 | 95575763 | G/A | TMEM56 | 0.7422 | 0.0296 | 0.0064 | 3.40 x 10^-6^ | 21.55 | 3.42 x 10^-4^ |
|  | rs149577802 | 3 | 163575879 | T/C | SLC2A2 | 0.0151 | -0.1088 | 0.0234 | 3.40 x 10^-6^ | 21.60 | 3.43 x 10^-4^ |
|  | rs2126371 | 16 | 82810664 | T/C | CDH13 | 0.3175 | -0.0289 | 0.0060 | 1.30 x 10^-6^ | 23.45 | 3.72 x 10^-4^ |
|  | rs3213829 | 10 | 61893898 | G/T | ANK3 | 0.5465 | 0.0260 | 0.0056 | 3.60 x 10^-6^ | 21.44 | 3.40 x 10^-4^ |
|  | rs692790 | 1 | 22044929 | C/T | USP48 | 0.8743 | 0.0404 | 0.0084 | 1.40 x 10^-6^ | 23.25 | 3.69 x 10^-4^ |
|  | rs74977546 | 2 | 72038130 | A/G | DYSF | 0.0529 | -0.0635 | 0.0127 | 5.50 x 10^-7^ | 25.10 | 3.98 x 10^-4^ |
| RBP4 | rs10882283 | 10 | 95360964 | C/A | RBP4 | 0.3701 | -0.1651 | 0.0273 | 1.38 x 10^-9^ | 36.57 | 1.10 x 10^-2^ |
|  | rs11084912 | 19 | 1859390 | C/A | KLF16 | 0.1760 | 0.1523 | 0.0321 | 2.14 x 10^-6^ | 22.51 | 6.77 x 10^-3^ |
|  | rs112357560 | 10 | 72575542 | A/G | SGPL1 | 0.0664 | -0.2332 | 0.0501 | 3.16 x 10^-6^ | 21.67 | 6.52 x 10^-3^ |
|  | rs115783813 | 5 | 49896349 | A/C | RP11-269M20.2 | 0.0676 | -0.241 | 0.0499 | 1.38 x 10^-6^ | 23.33 | 7.02 x 10^-3^ |
|  | rs117597613 | 18 | 30947875 | A/G | CCDC178 | 0.0291 | 0.3629 | 0.0767 | 2.24 x 10^-6^ | 22.39 | 6.74 x 10^-3^ |
|  | rs13173873 | 5 | 178649283 | A/G | ADAMTS2 | 0.2750 | -0.1323 | 0.0278 | 2.00 x 10^-6^ | 22.65 | 6.81 x 10^-3^ |
|  | rs143662949 | 3 | 10259317 | C/T | IRAK2 | 0.0118 | 0.6171 | 0.1195 | 2.40 x 10^-7^ | 26.67 | 8.01 x 10^-3^ |
|  | rs4697146 | 4 | 20164914 | T/C | MTCO3P44 | 0.6103 | -0.1221 | 0.026 | 2.51 x 10^-6^ | 22.05 | 6.64 x 10^-3^ |
|  | rs498422 | 6 | 32286761 | G/T | C6orf10 | 0.0583 | -0.2413 | 0.0525 | 4.17 x 10^-6^ | 21.12 | 6.36 x 10^-3^ |
|  | rs61864013 | 10 | 125169618 | A/G | RP11-282I1.1 | 0.0493 | 0.2779 | 0.0598 | 3.39 x 10^-6^ | 21.60 | 6.50 x 10^-3^ |
|  | rs6864862 | 5 | 76159445 | A/G | S100Z | 0.1962 | -0.1746 | 0.0333 | 1.55 x 10^-7^ | 27.49 | 8.26 x 10^-3^ |
|  | rs77691742 | 20 | 11924620 | T/G | BTBD3 | 0.8215 | -0.1513 | 0.0328 | 3.89 x 10^-6^ | 21.28 | 6.40 x 10^-3^ |
|  | rs8140446 | 22 | 49705242 | A/T | NA | 0.0664 | 0.2491 | 0.0512 | 1.12 x 10^-6^ | 23.67 | 7.12 x 10^-3^ |
| RDH16 | rs10808045 | 7 | 140180177 | C/T | MKRN1 | 0.6199 | -0.1277 | 0.0271 | 2.40 x 10^-6^ | 22.20 | 6.68 x 10^-3^ |
|  | rs114259703 | 4 | 40437075 | C/G | RBM47 | 0.0162 | 0.4802 | 0.0989 | 1.20 x 10^-6^ | 23.58 | 7.09 x 10^-3^ |
|  | rs138608414 | 10 | 56969277 | G/T | PCDH15 | 0.0189 | 0.4958 | 0.0968 | 3.02 x 10^-7^ | 26.23 | 7.88 x 10^-3^ |
|  | rs141738059 | 9 | 116832007 | C/T | AMBP | 0.0128 | 0.7819 | 0.1111 | 2.00 x 10^-12^ | 49.53 | 1.48 x 10^-2^ |
|  | rs148746153 | 17 | 10994293 | A/G | AC005284.1 | 0.0121 | -0.5647 | 0.1225 | 3.98 x 10^-6^ | 21.25 | 6.40 x 10^-3^ |
|  | rs181053633 | 1 | 90032656 | A/G | LRRC8B | 0.0125 | 0.5974 | 0.1234 | 1.29 x 10^-6^ | 23.44 | 7.05 x 10^-3^ |
|  | rs2242193 | 1 | 154995503 | A/G | DCST2 | 0.4177 | -0.1202 | 0.0255 | 2.40 x 10^-6^ | 22.22 | 6.69 x 10^-3^ |
|  | rs263123 | 6 | 142895127 | T/C | RP11-440G9.1 | 0.0403 | 0.3532 | 0.0667 | 1.17 x 10^-7^ | 28.04 | 8.42 x 10^-3^ |
|  | rs28406440 | 4 | 169754632 | G/C | PALLD | 0.0377 | 0.3086 | 0.0663 | 3.24 x 10^-6^ | 21.67 | 6.52 x 10^-3^ |
|  | rs4148324 | 2 | 234672722 | G/T | UGT1A4 | 0.3081 | 0.1987 | 0.0268 | 1.12 x 10^-13^ | 54.97 | 1.64 x 10^-2^ |
|  | rs4815933 | 20 | 6680319 | C/G | RP5-971N18.3 | 0.5890 | 0.1263 | 0.0269 | 2.57 x 10^-6^ | 22.04 | 6.63 x 10^-3^ |
|  | rs4936631 | 11 | 121326154 | G/C | SORL1 | 0.2533 | -0.1413 | 0.0292 | 1.29 x 10^-6^ | 23.42 | 7.04 x 10^-3^ |
|  | rs56061938 | 7 | 3061929 | T/G | CARD11 | 0.0928 | -0.2065 | 0.0438 | 2.34 x 10^-6^ | 22.23 | 6.69 x 10^-3^ |
|  | rs6103395 | 20 | 42240730 | A/G | IFT52 | 0.7363 | 0.1286 | 0.0282 | 4.90 x 10^-6^ | 20.80 | 6.26 x 10^-3^ |
|  | rs6595611 | 5 | 124748624 | T/C | LMNB1 | 0.8321 | -0.1799 | 0.0337 | 9.12 x 10^-8^ | 28.50 | 8.56 x 10^-3^ |
|  | rs7249489 | 19 | 41978596 | A/C | PCAT19 | 0.2498 | -0.1334 | 0.0291 | 4.57 x 10^-6^ | 21.01 | 6.33 x 10^-3^ |
|  | rs76552190 | 14 | 28430652 | C/G | AL445384.1 | 0.0217 | 0.4267 | 0.0915 | 3.16 x 10^-6^ | 21.75 | 6.54 x 10^-3^ |
|  | rs76603521 | 2 | 124874290 | C/A | CNTNAP5 | 0.1344 | 0.1827 | 0.0366 | 5.75 x 10^-7^ | 24.92 | 7.49 x 10^-3^ |
| CRABP1 | rs10402311 | 19 | 55065158 | C/T | AC009892.2 | 0.2652 | 0.1346 | 0.0281 | 1.66 x 10^-6^ | 22.94 | 6.90 x 10^-3^ |
|  | rs115624143 | 4 | 64908870 | A/C | DPP3P1 | 0.0076 | -0.6679 | 0.143 | 3.02 x 10^-6^ | 21.81 | 6.57 x 10^-3^ |
|  | rs11611699 | 12 | 14351518 | T/C | RP11-298E10.1 | 0.0589 | -0.2612 | 0.0526 | 6.92 x 10^-7^ | 24.66 | 7.41 x 10^-3^ |
|  | rs12194933 | 6 | 159641706 | A/G | FNDC1 | 0.1365 | 0.177 | 0.0366 | 1.38 x 10^-6^ | 23.39 | 7.04 x 10^-3^ |
|  | rs142517401 | 3 | 66161137 | G/T | SLC25A26 | 0.1145 | 0.1967 | 0.0401 | 9.55 x 10^-7^ | 24.06 | 7.24 x 10^-3^ |
|  | rs167189 | 3 | 139295138 | A/G | NMNAT3 | 0.6752 | -0.1232 | 0.0267 | 3.89 x 10^-6^ | 21.29 | 6.41 x 10^-3^ |
|  | rs28477514 | 21 | 32747183 | C/T | TIAM1 | 0.1440 | 0.1615 | 0.035 | 3.98 x 10^-6^ | 21.29 | 6.41 x 10^-3^ |
|  | rs550880546 | 3 | 176132701 | T/A | U8 | 0.0211 | 0.4329 | 0.0913 | 2.14 x 10^-6^ | 22.48 | 6.76 x 10^-3^ |
|  | rs55683390 | 3 | 3538689 | A/T | AC026188.1 | 0.6762 | -0.1283 | 0.0273 | 2.69 x 10^-6^ | 22.09 | 6.65 x 10^-3^ |
|  | rs570618 | 1 | 196657064 | G/T | CFH | 0.6115 | -0.1342 | 0.0254 | 1.23 x 10^-7^ | 27.91 | 8.39 x 10^-3^ |
|  | rs58015908 | 3 | 13866984 | T/C | WNT7A | 0.0291 | -0.3371 | 0.0738 | 4.90 x 10^-6^ | 20.86 | 6.28 x 10^-3^ |
|  | rs6505079 | 17 | 26718177 | A/T | SARM1 | 0.5107 | 0.2268 | 0.0242 | 8.13 x 10^-21^ | 87.83 | 2.59 x 10^-2^ |
|  | rs72785161 | 16 | 68751154 | T/C | CDH3 | 0.0741 | -0.2343 | 0.0483 | 1.23 x 10^-6^ | 23.53 | 7.08 x 10^-3^ |
|  | rs74480769 | 5 | 40972211 | G/A | C7 | 0.0317 | -0.532 | 0.0732 | 3.63 x 10^-13^ | 52.82 | 1.57 x 10^-2^ |
|  | rs7847938 | 9 | 73111175 | C/T | TRPM3 | 0.4650 | 0.1205 | 0.0246 | 9.33 x 10^-7^ | 23.99 | 7.22 x 10^-3^ |
|  | rs9485099 | 6 | 146906650 | T/G | RP11-15G8.1 | 0.2971 | 0.1285 | 0.0275 | 3.02 x 10^-6^ | 21.83 | 6.57 x 10^-3^ |

Abbreviations: Chr, chromosome; CRABP1, Cellular retinoic acid-binding protein 1; EA, effect allele; EAF, effect allele frequency; OA, other allele; RBP4, Retinol-binding protein 4; RDH16, Retinol dehydrogenase 16; SE, standard error. SNP, single nucleotide polymorphism.
